# Saturated Alcohols Electrocatalytic Oxidations on Ni-Co Bimetal Oxide Featuring Balanced B- and L-Acidic Active Sites

**DOI:** 10.1007/s40820-025-01893-z

**Published:** 2025-08-25

**Authors:** Junqing Ma, Wenshu Luo, Xunlu Wang, Xu Yu, Jiacheng Jayden Wang, Huashuai Hu, Hanxiao Du, Jianrong Zeng, Wei Chen, Minghui Yang, Jiacheng Wang, Xiangzhi Cui

**Affiliations:** 1 State Key Laboratory of High-Performance Ceramics and Superfine Microstructure, Shanghai Institute of Ceramics Chinese Academy of Sciences, Shanghai, 200050 People’s Republic of China; 2 Center of Materials Science and Optoelectronics Engineering, University of Chinese Academy of Sciences, Beijing, 100049 People’s Republic of China; 3 Zhejiang Key Laboratory for Island Green Energy and New Materials, Institute of Electrochemistry, School of Materials Science and Engineering, Taizhou University, Taizhou, 318000 Zhejiang People’s Republic of China; 4 School of Environmental Science and Technology, Dalian University of Technology, Dalian, 116024 People’s Republic of China; 5 Shanghai Synchrotron Radiation Facility, Shanghai Advanced Research Institute Chinese Academy of Sciences, Shanghai, 201204 People’s Republic of China; 6 Department of Materials Design and Innovation, University at Buffalo The State University of New York, Buffalo , NY 14260 USA; 7 School of Chemistry and Materials Science, Hangzhou Institute for Advanced Study University of Chinese Academy of Sciences, Hangzhou, 310024 People’s Republic of China

**Keywords:** Solid-acid electrocatalyst, Alcohol oxidation reaction, Brønsted acid sites, Lewis acid sites; C_1_-C_6_ saturated alcohols

## Abstract

Investigating structural and hydroxyl group effects in electrooxidation of alcohols to value-added products by solid-acid electrocatalysts is essential for upgrading biomass alcohols. Herein, we report efficient electrocatalytic oxidations of saturated alcohols (C_1_-C_6_) to selectively form formate using NiCo hydroxide (NiCo–OH) derived NiCo_2_O_4_ solid-acid electrocatalysts with balanced Lewis acid (LASs) and Brønsted acid sites (BASs). Thermal treatment transforms BASs-rich (89.6%) NiCo–OH into NiCo_2_O_4_ with nearly equal distribution of LASs (53.1%) and BASs (46.9%) which synergistically promote adsorption and activation of OH^−^ and alcohol molecules for enhanced oxidation activity. In contrast, BASs-enriched NiCo–OH facilitates formation of higher valence metal sites, beneficial for water oxidation. The combined experimental studies and theoretical calculation imply the oxidation ability of C_1_-C_6_ alcohols increases as increased number of hydroxyl groups and decreased HOMO–LUMO gaps: methanol (C_1_) < ethylene glycol (C_2_) < glycerol (C_3_) < meso-erythritol (C_4_) < xylitol (C_5_) < sorbitol (C_6_), while the formate selectivity shows the opposite trend from 100 to 80%. This study unveils synergistic roles of LASs and BASs, as well as hydroxyl group effect in electro-upgrading of alcohols using solid-acid electrocatalysts.

**Supplementary Information:**

The online version contains supplementary material available at 10.1007/s40820-025-01893-z.

## Highlights

- NiCo–OH has a relatively high Brønsted acid sites (BASs) content (89.6%), which can promote the adsorption of OH^−^ and inhibit the co-adsorption of OH^−^ and alcohols, resulting in poor alcohol oxidation reaction (AOR) activity but higher oxygen evolution reaction activity.
- NiCo–OH-derived NiCo_2_O_4_ solid-acid electrocatalysts with balanced BASs (46.9%) and Lewis acid sites (53.1%) facilitates co-adsorption of alcohols molecules and OH^−^, thereby favoring the AOR.
- In the AOR on NiCo_2_O_4_, as the number of hydroxyl groups in C_1_-C_6_ saturated alcohols increases, the activity shows an increasing trend: C_1_<C_2_<C_3_<C_4_<C_5_<C_6_.

**Supplementary Information:**

The online version contains supplementary material available at 10.1007/s40820-025-01893-z.

## Graphical abstract

**Figure:**
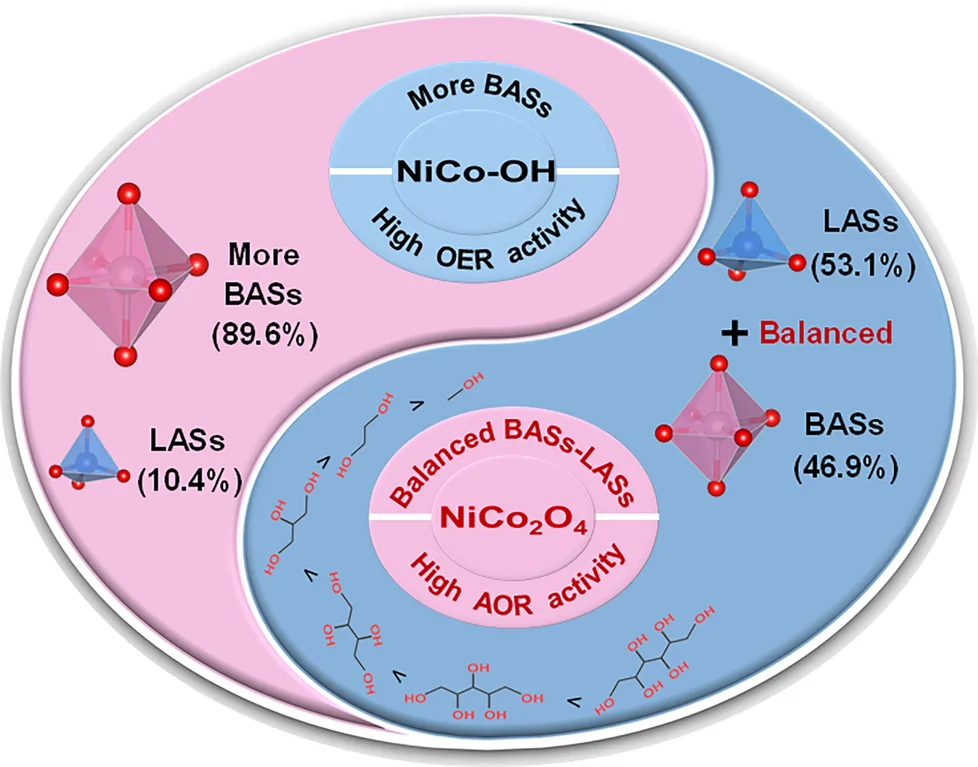

## Introduction

The electrocatalytic oxidations of renewable bioenergy into high-value chemicals aligns with the concepts of sustainable development and environmental friendliness [1–3]. Biomass alcohols, such as glycerol [4–6], xylitol [7], and sorbitol [8] are particularly noteworthy as potential raw materials. They are biomass-derived platform molecules (BDPMs) proposed by the U.S. Department of Energy in 2010 as part of the “TOP 10 + 4” BDPMs that can be used for synthesizing high-value chemicals and fuels [9]. Not only have they abundant resources and low costs, but also are easily oxidized due to the presence of reactive hydroxyl groups in their molecules. For example, Bambagioni et al. discovered that the electrooxidation of renewable alcohols can achieve up to 67% energy and water savings compared to traditional methods when producing an equivalent amount of hydrogen [10]. Additionally, the electrochemical oxidation of renewable alcohols can convert biomass feedstocks into transportable fuels and high-value-added fine chemicals such as formic acid, glycolic acid, and glyceric acid, etc. using renewable energy electricity [11–14]. Therefore, electrooxidation of renewable alcohols (e.g., methanol, ethanol, and glycerol) have been extensively investigated in recent years.

Nickel-based and cobalt-based electrocatalysts are widely utilized in large-scale industrial water electrolysis and organic electrooxidation (see Tables S1, S2 and Fig. S1 for details) [15–20]. For instance, Chen et al. found that doping cobalt into nickel hydroxide (NiCo hydroxide) promotes the intercalation and deintercalation of protons and oxygen anions at the catalyst surface, thereby exhibiting enhanced glycerol electrooxidation activity [21]. In our previous work, we found that the solid-acid electrocatalyst NiCo_2_O_4_ on nickel foam shows synergistic Lewis acid sites (LASs) and Brønsted acid sites (BASs), achieving high-efficiency glycerol electrooxidation [22]. However, systematic studies on the mechanisms of alcohol oxidation reaction (AOR) are still lacking by changing the ratio of LASs and BASs in transition metal-based hydroxides and oxides solid-acid electrocatalysts. Also, identifying and elucidating oxidation mechanisms of various alcohols by solid-acid electrocatalysts are crucial for further guiding the design of high-activity electrocatalysts.

Despite extensive efforts to enhance the AOR activity of cobalt-based and nickel-based electrocatalysts by various strategies, little attention has been devoted to the intrinsic properties of alcohol molecules themselves. The electrochemical AORs typically involve hydroxyl group transformations and carbon–carbon bond cleavages. The position and quantity of hydroxyl groups in alcohol molecules could influence the activity of AORs. For example, on gold and platinum electrodes, it has been observed that polyols exhibit higher activity compared to monohydric alcohols [23]. Furthermore, the effect of hydroxyl group positioning has also been documented. For instance, on noble metals, 2,3-butanediol (2,3-BD) shows higher reactivity toward Au compared to 1,3-butanediol (1,3-BD) and 1,4-butanediol (1,4-BD), potentially due to the strong resonance effect of the hydroxyl group in 2,3-BD [24]. For non-noble metal Co_3_O_4_ electrocatalyst, the impact of hydroxyl group positioning in butanediol electrooxidation under alkaline conditions was studied, showing that closer proximity of hydroxyl groups enhances reactivity, with vicinal butanediols exhibiting higher oxidation propensity (1,2-BD > 2,3-BD > 1,3-BD > 1,4-BD) [25]. However, to the best of our knowledge, a systematic study of influence mechanism of hydroxyl group quantity in saturated alcohols on non-noble metal oxide electrooxidation behavior remains lacking. Given the potential of multiple oxidation reactions on polyhydric saturated alcohols, studying and leveraging the priorities and mechanisms behind hydroxyl group transformations and selective C–C bond cleavages holds significant importance for enhancing the value-added products in electrochemical synthesis targets.

**Fig. 1:**
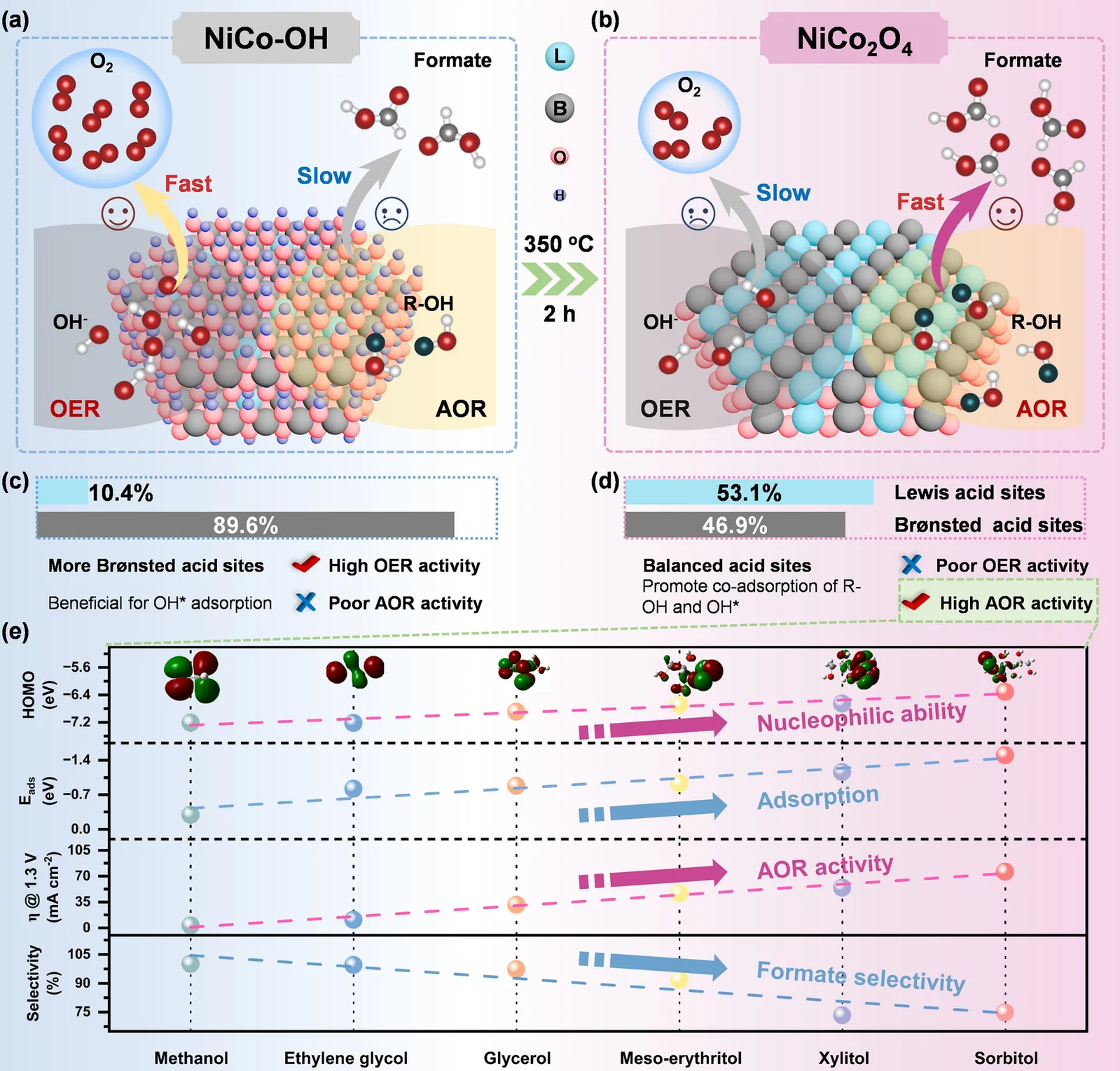
Thermal transformation of NiCo–OH with less LASs and more BASs into NiCo_2_O_4_ with balanced BASs and LASs for efficient AOR. Schematic diagram of **a** NiCo–OH showing fast OER, but slow AOR process and **b** NiCo–OH–derived NiCo_2_O_4_ presenting slow OER, but fast AOR process. Bar charts of the relative ratio of LASs and BASs in **c** NiCo–OH and **d** NiCo_2_O_4_. **e** Comparison of the nucleophilic ability, adsorption ability, AOR activity and formate selectivity of C_1_-C_6_ saturated alcohols including methanol, ethylene glycerol, glycerol, meso-erythritol, xylitol, and sorbitol on NiCo_2_O_4_ solid-acid electrocatalysts with balanced BASs and LASs

Herein, we report electrocatalytic oxidations of C_1_-C_6_ saturated alcohols to selectively produce high-valued formate using NiCo hydroxide (NiCo–OH) derived NiCo_2_O_4_ solid-acid electrocatalyst with balanced BASs and LASs (Fig. 1a-d). Experimental results and theoretical calculations demonstrated that with a higher proportion of BASs (89.6%, corresponding to high-valence Ni and Co sites), NiCo–OH is more readily activated to form high-valence NiOOH and CoOOH, thereby enhancing the oxygen evolution reaction (OER) activity (Fig. 1a, c). Conversely, the thermally treated oxide NiCo_2_O_4_ possesses a nearly equal proportion of LASs (53.1%) and BASs (46.9%), which better facilitate OH* formation and alcohol molecule adsorption, thereby exhibiting excellent AOR activity (Fig. 1b, d). Furthermore, the quantity effect of hydroxyl groups in alcohols on the electrooxidation activity by NiCo_2_O_4_ solid-acid electrocatalysts was systematically studied using a series of saturated alcohols for the first time including methanol, ethylene glycol, glycerol, meso-erythritol, xylitol, and sorbitol (Fig. 1e). The nucleophilic ability, adsorption energy and activity of alcohol molecules on the surface of NiCo_2_O_4_ are enhanced with the increase of the number of hydroxyl groups in saturated alcohols; the selectivity of formate gradually decreases. These results indicate that there is a close correlation between the intrinsic properties of alcohol molecules and their electrocatalytic performance for non-noble metal catalysts. This work provides deep insights into the effect of LASs and BASs, as well as provide guidance for designing efficient electrocatalytic biomass reactions.

## Experimental Section

### Materials

Methanol (CH_3_OH, CP), ethylene glycol (C_2_H_6_O_2_, AR), glycerol (C_3_H_8_O_3_, AR), potassium hydroxide (KOH, AR), and hydrochloric acid (HCl, AR, 36%–38%) were purchased from Sinopharm Chemical Reagent Co., Ltd. (Shanghai, China). Meso-erythritol (C_4_H_10_O_4_, 99%, Adamas-beta), xylitol (C_5_H_12_O_5_, 99% + , Adamas-beta), D-sorbitol (C_6_H_14_O_6_, 98% + , Adamas-beta), formic acid (HCOOH, RG, Adamas), 2,3-dihydroxypropanoic acid (C_3_H_6_O_4_, RG, Adamas), glycolic acid (C_2_H_4_O_3_, RG, Adamas), acetic acid (CH_3_COOH, RG, Adamas), lactic acid (C_3_H_6_O_3_, RG, Adamas), oxalic acid (C_2_H_2_O_4_, RG, Adamas), cobalt nitrate hexahydrate (Co(NO_3_)_2_·6H_2_O, 99 wt%, Adamas-beta, RG), nickel nitrate hexahydrate (Ni(NO_3_)_2_·6H_2_O, 99 wt%, Adamas-beta, RG) and nafion (ALORICH) were purchased from Shanghai Titan Scientific Co., Ltd. (Shanghai, China). Nickel foam (1 mm thick) was purchased from Saibo Electrochemistry (Beijing, China). Ethanol (AR) was purchased from Shanghai Lingfeng Chemical Reagent Co., Ltd. Carbon felt (Carbon energy) was purchased from Suzhou Shengernuo Technology Co., Ltd. Commercial Pt/C (40 wt%) was purchased from Shanghai He-Sen Electric Co. Ltd. (Shanghai, China). All materials were used without treatment.

### Preparation of NiCo Hydroxide (NiCo-OH) on Nickel Foam

The preparation of NiCo–OH electrocatalyst is detailed in the previous work [22]. Specifically, a piece of cleaned nickel foam (NF) was utilized as the working electrode, Ag/AgCl (3 M KCl) was used as the reference electrode, and carbon rod was used as the counter electrode. A solution containing 30 mM Co(NO_3_)_2_·6H_2_O and 30 mM Ni(NO_3_)_2_·6H_2_O dissolved in 100 mL of deionized water was prepared for electrodeposition. The electrodeposition process was carried out at a constant voltage of −1 V vs. Ag/AgCl (3 M KCl) for 600 s. Afterward, the working electrode was removed and rinsed with deionized water three times to obtain NiCo–OH electrocatalyst through subsequent drying.

### Preparation of NiCo_2_O_4_ on Nickel Foam

NiCo_2_O_4_ was obtained by heat treatment of NiCo–OH in air at 300 °C for 3 h at a heating rate of 1 °C min^−1^.

### Material Characterization

The surface morphology of the samples was characterized by scanning electron microscope (SEM, FEI Magellan 400). Transmission electron microscopy (TEM), high-resolution TEM (HRTEM) and energy-dispersive X-ray spectroscopy (EDS) mapping were carried out on Titan G2 60–300 Cs-corrected TEM. The crystalline structure and purity of the samples were characterized by X-ray diffraction (XRD, D8 ADVANCE, Cu Kα radiation) with testing conditions set at a scanning speed of 4° min^−1^ and a range of 10°—80°. Ex situ Raman spectroscopy was acquired on LabRAM HR Evolution Lab-HRDLS 20 instrument with an excitation wavelength of 532 nm. The Ni foam supported NiCo–OH or NiCo_2_O_4_, Pt wire, and Ag/AgCl were used as the working, counter, and reference electrodes, respectively. The composition and electronic structure of the obtained samples were analyzed using X-ray photoelectron spectroscopy (XPS, Thermo ESCALAB250xi) and X-ray absorption fine structure (XAFS) which were acquired from the beamline BL13SSW at Shanghai Synchrotron Radiation Facility. In order to effectively avoid the interference of the NF, the catalyst material was electrodeposited on the CF substrate for XAFS testing. The detection of hydroxyl radicals was accomplished by using 5,5-dimethyl-1-pyrroline-N-oxide (DMPO) as a radical scavenger on Bruker A300 electron spin resonance (ESR) spectrometer.

Electrochemical in-situ Raman spectroscopy measurements were carried out on an EC-RAIR-H Raman spectrometer equipped with a 532 nm laser and an electrochemical workstation. The electrolytic cell was made of polytetrafluoroethylene. The sample area was exposed to the laser beam through a quartz window. Meanwhile, the self-supported electrode was embedded in the electrolytic cell, and its surface was ensured to be perpendicular to the incident laser to achieve optimal contact. The prepared catalyst was used as the working electrode, a platinum wire and Ag/AgCl were used as the counter electrode and the reference electrode, respectively. The electrolyte was a 1 mol L^−^^1^ KOH solution containing 0.1 mol L^−1^ methanol. Raman spectra were recorded after reacting for 300 s at different potentials.

Pyridine monitored by Fourier Transform infrared spectroscopy (FTIR) were performed using a FTIR-650 spectrometer, in a home-made vacuum infrared cell with CaF_2_ windows, a self-supporting wafer of the sample was initially dried under vacuum at 150 °C for 1 h, and then cooled down to 50 °C. Afterward, the wafer was saturated with about 25 mbar of pyridine vapor at 50 °C for 10 min and then evacuated again for 30 min to fully remove physisorbed pyridine. Finally, the evacuated sample containing chemisorbed pyridine was subjected to TPD 150 °C for 30 min, with a heating rate of 10 °C min^−1^, and the iR spectra were recorded in-situ at these temperatures. The amounts of acid sites were determined from the integral intensity of characteristic bands (1450 cm^−1^ for Lewis acid sites, and 1540 cm^−1^ for Brønsted acid sites) using the molar extinction coefficients of Emeis [26].

Quantification Method: LASs/BASs ratios were calculated using:

1 $$C_{BASs} = 1.88I_{A(BASs)} R2/W$$

2 $$C_{LASs} = 1.42I_{A(LASs)} R2/W$$

3 $$proportion \, \left( \% \right) \, = \frac{{C_{LASs/BASs} }}{{C_{LASs} + C_{BASs} }} \times 100$$

*C* represents the concentration of pyridine adsorbed at the acid sites of acid B and acid L (mmol g^−1^). *I*_A_ denotes the corresponding peak areas. 1.88 and 1.42 are the extinction coefficients of BASs and LASs respectively. *R* represents the radius of the tablet (cm). W represents the mass of the tablet (mg).

### Electrochemical Measurements

All OER and AOR half-reactions were performed using a standard three-electrode system (provided by Jiangsu Boke New Materials Technology Co., Ltd) with a CHI 760E electrochemical workstation at 25 °C. The as-prepared NF (1 × 1 cm^2^) supported NiCo–OH or NiCo_2_O_4_ was directly utilized as the working electrode, and a Hg/HgO and carbon rod were respectively used as the reference and the counter electrode. 1 M KOH and 1 M KOH containing 0.1 M alcohol was used as electrolyte for OER and AOR, respectively. The cyclic voltammetry (CV) curves and linear sweep voltammetry (LSV) experiments were performed within the potential range of 0–0.8 V with a scan rate of 100 and 5 mV s^−1^, respectively. Notably, all CV and LSV curves were performed without iR correction. In the pH-dependent test, KOH solutions with concentrations of X M (where X is 0.1, 0.5, 0.8, 1.0, and 2.0 respectively) and 0.1 M of methanol, ethylene glycol, glycerol, meso-erythritol, xylitol or sorbitol were selected as electrolyte solutions for the test. The electrochemical impedance spectroscopy (EIS) was conducted in a frequency range from 0.01–10^5^ Hz with 5 mV amplitude. Operando EIS measurements were conducted over a frequency range from 0.1 to 10^4^ Hz. The potentials versus Hg/HgO were converted to the reversible hydrogen potential (RHE) by applying the following equation: E_(RHE)_ = E_(Hg/HgO)_ + 0.098 + 0.059 × PH. CV curves at different scan speeds (10, 20, 40, 60, 80, 100, and 120 mV s^−1^) were conducted in the potential range of 0.05–0.15 V (vs. Hg/HgO) to calculate the double-layer capacitance (*C*_dl_).

### Analysis of Near-Surface pH

First, a solution with a pH of approximately 13 was prepared by mixing 0.1 M KOH with 0.1 M alcohol (methanol, ethylene glycol, glycerol, meso-erythritol, xylitol, or sorbitol). The actual pH value of the solution was then precisely measured using a calibrated high-accuracy pH meter to verify system consistency. Subsequently, i-t test was conducted in a standard three-electrode configuration under an applied potential of 1.5 V vs. RHE. During the test, a Shanghai Sanxin LabSen series Swiss-process high-precision micro-glass pH composite electrode probe is adopted to monitor the pH change of the solution on the catalyst surface in real time, thereby quantitatively analyzing the consumption of OH^−^ ions during the reaction process.

### Products Analysis

The product analysis was conducted using high performance liquid chromatography (HPLC), which was equipped with a Coregel-87H3 column on Agilent LC2050. The specific testing procedure shows as following:

To avoid the impact of the counter electrode on product analysis, a three-electrode system was assembled to perform i-t testing in an H-shaped cell. One side consisted of the counter electrode (carbon rod), and the electrolyte used was 1 M KOH (40 mL). On the other side, there were the working electrode (catalysts measuring 1 × 1 cm^2^) and reference electrode (Hg/HgO electrode), with an electrolyte comprising 1 M KOH containing 0.1 M alcohol (40 mL). After a certain period of testing (1, 2, 4, and 6 h), 600 μL of electrolyte from the working electrode side was extracted and neutralized by 600 μL of 5 mM H_2_SO_4_ until pH below 7. The eluent employed was H_2_SO_4_, while the temperature of the column oven was maintained at 60 °C. To ensure optimal product separation, all samples were subjected to analysis using two detectors. Firstly, an Ultraviolet detector (UV) operating at a wavelength of 210 nm and a flow rate of 0.6 mL min^−1^ was employed to separate products exhibiting UV absorption characteristics such as formic acid, oxalic acid, glyceric acid, glycolic acid, lactic acid, and acetic acid. Additionally, a refractive index detector (RID) was utilized to effectively separate various alcohols lacking UV absorption properties including C_1_-C_6_ saturated alcohols. The selectivity (S) of the products are calculated by the following equations:

4 $$S\left( \% \right)\, = \,\frac{{concentration\;of\;the\;product}}{{concentration\;of\;all\;products}} \times 100\%$$

### Computational Details

*Ab initial calculations*: The adsorption Gibbs free energy in this study was computed through density functional theory (DFT) calculations using the Vienna ab initio Simulation Package (VASP) [27]. The exchange–correlation potential is described by using the generalized gradient approximation Perdew-Burke-Ernzerh (GGA-PBE) method [28]. The plane-wave cut-off energy of 450 eV was employed. In geometry optimization, all samples were fully optimized until the total energy converged to 10^–5^ eV and force on each atom converged to less than 0.05 eV Å^−1^. Gamma Scheme k-points of 3 × 3 × 1 were applied for all the surface calculations. The DFT-D3 empirical correction method was employed to describe van der Waals interactions. Atoms at bottom were fixed in all the calculations. The NiCo_2_O_4_ (110) surface was selected based on its stability and prevalence in spinel oxide. The (001) surface was selected for the NiCo–OH model.

*Quantum chemistry calculations:* The quantum chemistry calculations of the alcohol molecules including complete geometrical optimization were performed using B3LYP hybrid density functional with 6–31 G (d, p) basis set of Gaussian 09 W. The HOMO and LUMO were analyzed and plotted by the graphical interface of GaussView5.0.

## Results and Discussion

### Synthesis and Structural Characterizations of NiCo-Based Solid-Acid Electrocatalysts

**Fig. 2:**
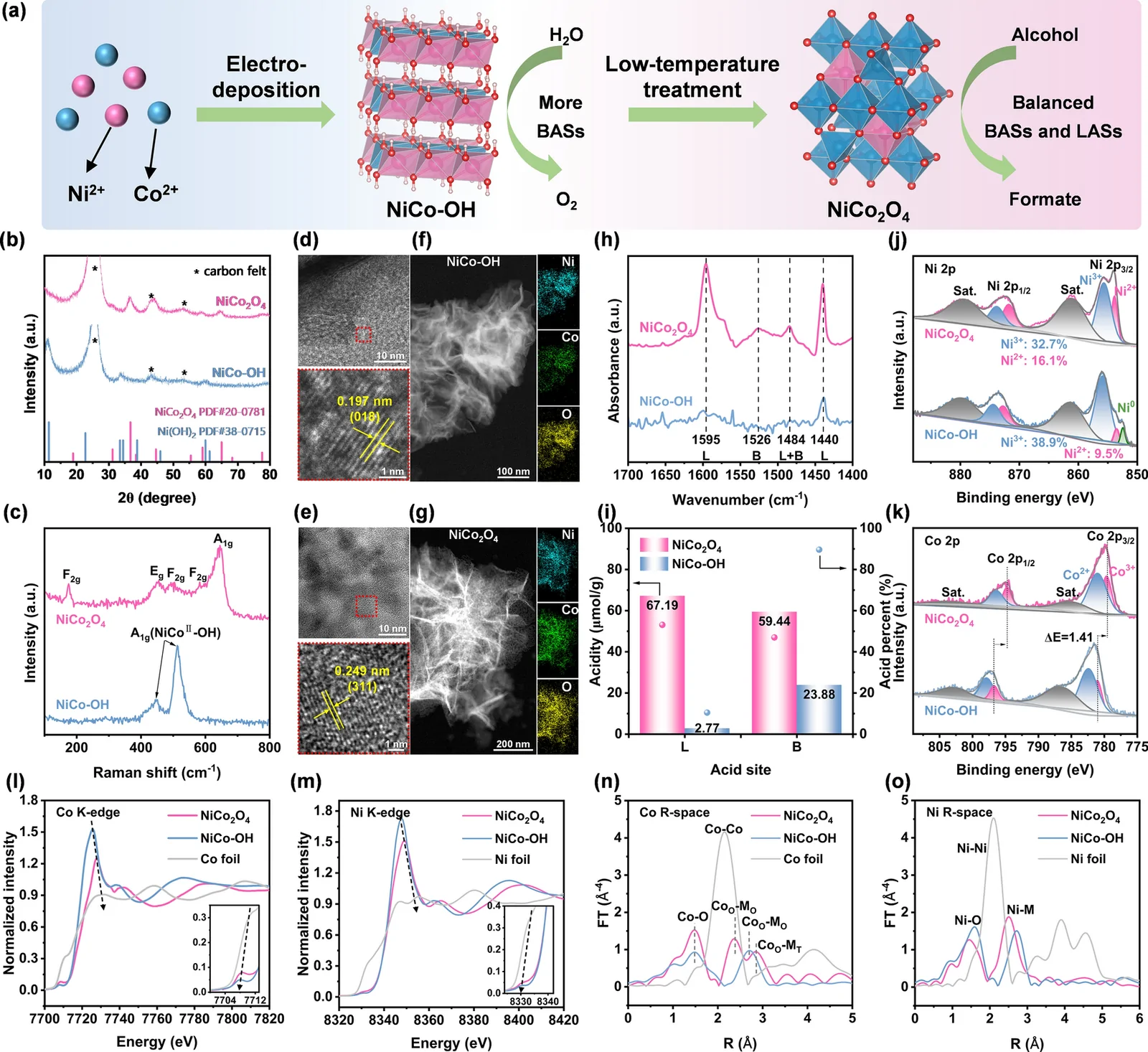
Preparation and structural characterizations of NiCo–OH and NiCo_2_O_4_ solid-acid electrocatalysts. **a** Schematic illustration of synthesis procedure by electrodeposition and subsequent calcination. **b** XRD patterns and **c** Raman spectra of NiCo–OH and NiCo_2_O_4_. HRTEM, HAADF, and elemental mapping images of **d, f** NiCo–OH and **e, g** NiCo_2_O_4_. **h** FTIR spectra of NiCo–OH and NiCo_2_O_4_ after pyridine adsorption. L: LASs; B: BASs. **i** Histograms of acidity and corresponding ratios of LASs and BASs in NiCo–OH and NiCo_2_O_4_. High-resolution XPS spectra for NiCo–OH and NiCo_2_O_4_ in the **j** Ni 2*p* and **k** Co 2*p* regions. XANES of **l** Co K-edge and **m** Ni K-edge of NiCo–OH and NiCo_2_O_4_ with the corresponding enlarged parts in the insets. EXAFS of **n** Co K-edge and **o** Ni K-edge in the R-space of NiCo–OH and NiCo_2_O_4_

The route for synthesizing NiCo-based solid-acid electrocatalysts is depicted in Fig. 2a (details in the experimental section). The layered NiCo hydroxide (NiCo–OH) nanosheets were synthesized via an electrodeposition method, followed by thermal treatment to obtain spinel-structured NiCo_2_O_4_. The crystal structures of two catalysts were analyzed using X-ray diffraction (XRD), as shown in Fig. 2b. To mitigate interference from the nickel foam (NF) substrate, carbon felt (CF) was used as the substrate for XRD analysis (Fig. S2). The diffraction peaks of NiCo–OH were observed at 11.1°, 33.6°, and 46.0°, corresponding to the (003), (101), and (018) crystal planes and can be well assigned to Ni(OH)_2_ (PDF# 38–0715) [29]. Upon heat treatment, distinct diffraction peaks appeared at 36.7°, 44.6°, and 64.9° for NiCo_2_O_4_ corresponding to its (003), (400), and (440) lattice planes with a cubic and spinel structure with *Fd-3m* space group according to PDF# 20–0781 of NiCo_2_O_4_ [30]. Raman spectroscopy further confirms this structural transformation. As shown in Fig. 2c**,** Raman spectroscopy of NiCo–OH exhibits two characteristic peaks in the range of 100–800 cm^−1^, corresponding to the Raman band of A_1g_ and was assigned to trivalent Ni and Co. In sharp contrast, NiCo–OH-derived NiCo_2_O_4_ has five Raman band modes in the range of 0–800 cm^−1^: A_1g_, F_2g_, F_2g_, E_g_, and F_2g_. Scanning electron microscopy (SEM) images show that both NiCo–OH and NiCo_2_O_4_ nanosheets densely and uniformly cover the surface of NF (Figs. S3 and S4). Their nanosheet-like morphologies were confirmed by transmission electron microscopy (TEM) (Figs. S5 and S6). The high-resolution TEM (HRTEM) characterizations reveal their crystalline natures with lattice spacing distances of 1.97 Å for NiCo–OH and 2.49 Å for NiCo_2_O_4_ (Fig. 2d, e), which correspond to the (018) and (311), consistent with the XRD results. XRD and Fast Fourier transform images (Fig. S7) proved that the crystallinity of the material was relatively low. However, this characteristic provided more potential active sites for the subsequent electrocatalytic reaction. Additionally, the high-angle annular dark field (HAADF) image and corresponding energy-dispersive spectroscopy (EDS) mapping demonstrate the homogeneous distribution of Ni, Co, and O elements in NiCo–OH and NiCo_2_O_4_ nanosheet (Fig. 2f, g).

In addition, LASs and BASs in NiCo–OH and NiCo_2_O_4_ electrocatalysts were analyzed by recording the Fourier transform infrared spectroscopy (FTIR) spectra using pyridine adsorption (Fig. 2h). In general, the adsorption of pyridine molecules at LASs gives rise to infrared activity bands at 1440, 1484, and 1595 cm^−1^, while BASs at 1484 and 1526 cm^−1^ [31, 32] The results summarizing the number and proportion of LASs and BASs are presented in Fig. 2i and Table S3. The NiCo–OH surfaces possess significant abundance of BASs (89.6%), while NiCo_2_O_4_ has a balanced proportion of LASs (53.1%) and BASs (46.9%), beneficial for spontaneous activation of OH^−^ and alcohol molecules during AOR.

The X-ray photoelectron spectroscopy (XPS) technique was further employed to investigate acid sites environments of Ni and Co in NiCo–OH and NiCo_2_O_4_ [33]. The survey spectra of both NiCo–OH and NiCo_2_O_4_ show distinct Ni, Co, and O signals (Figs. S8 and S9). As shown in Fig. 2j, the high-resolution Ni 2*p* spectra were deconvoluted into three spin–orbit peaks, corresponding to the Ni^2+^ signals (853.9 and 872.3 eV), Ni^3+^ signals (855.8 and 874.9 eV), and satellite peaks signals (861.1 and 879.7 eV) [34–36]. Notably, the proportion of Ni^3+^ in NiCo_2_O_4_ (32.7%) is relatively lower than that observed in NiCo–OH (38.4%). In the NiCo–OH sample, distinct Ni^0^ 2*p* signals are clearly observed, with characteristic peaks located at 852.4 and 870.4 eV, primarily originating from the metallic Ni phase in the NF substrate [37, 38]. Furthermore, the high-resolution XPS spectrum reveals Co 2*p* peaks at binding energies of 779.6 and 794.7 eV, 781.0 and 796.4 eV, 784.8 and 803.7 eV, which can be indexed to Co^3+^, Co^2+^, and satellite peaks, respectively [39–41]. The presence of Co^3+^ in NiCo_2_O_4_ is observed at a lower binding energy with a shift of 1.41 eV (Fig. 2k). This observation confirms the higher oxidation state of Ni and Co in NiCo–OH. Therefore, the XPS results indicate a higher abundance of BASs (Ni^3+^ and Co^3+^) in NiCo–OH, with the formation of NiCo_2_O_4_ occurring upon heat treatment resulting in a reduced overall valence state and a balanced distribution of LASs and BASs. The high-resolution O 1 s XPS spectrum exhibits three peaks located at 529.5, 531.0, and 532.5 eV (Fig. S10), corresponding to lattice oxygen, hydroxyl, and adsorbed H_2_O, respectively [42, 43]. The lattice oxygen ratio in NiCo_2_O_4_ is observed to reach 41.6%, significantly surpassing 4% in NiCo–OH.

Normalized X-ray absorption near-edge structure (XANES) and extended X-ray absorption fine structure (EXAFS) spectra were used to further characterize coordination structures of NiCo–OH and NiCo_2_O_4_. In Fig. 2l, the absorption edge energy of Co K-edge in NiCo–OH is similar to that of NiCo_2_O_4_, but exhibits higher white line intensity compared to NiCo_2_O_4_, indicating a slightly higher average state of Co element in NiCo–OH compared to NiCo_2_O_4_. The magnified region showing a higher intensity of the pre-edge peak in NiCo_2_O_4_ also indicates the existence of more Co^2+^. Similarly, the valence state of Ni in NiCo–OH is slightly higher than that in NiCo_2_O_4_ (Fig. 2m). This result is consistent with the XPS results, suggesting the presence of more BASs in NiCo–OH and relatively more LASs in NiCo_2_O_4_ [44, 45]. The corresponding Fourier transform data of the Co and Ni K-edge are also analyzed the electronic structure of the catalysts has undergone alterations before and after annealing. In Fig. 2n, o, Co exclusively occupies octahedral sites (Co_O_-M_O_) in NiCo–OH, whereas in NiCo_2_O_4_, it is present in both octahedral (Co_O_-M_O_) and tetrahedral sites (Co_O_-M_T_). Additionally, Ni is situated at octahedral sites in both NiCo–OH and NiCo_2_O_4_ [46].

Overall, the presence of a greater number of hypervalent species in the hydroxides, i.e., more BASs could be crucial for improving OER kinetics. However, following thermal transformation, a balanced proportion of LASs and BASs could be achieved in NiCo_2_O_4_ solid-acid electrocatalyst, more favorable for promoting the AOR activity through synergistic activation of OH^−^ and alcohol molecules.

### Analyzing MOR and OER Activity of NiCo-Based Solid-Acid Electrocatalysts

**Fig. 3:**
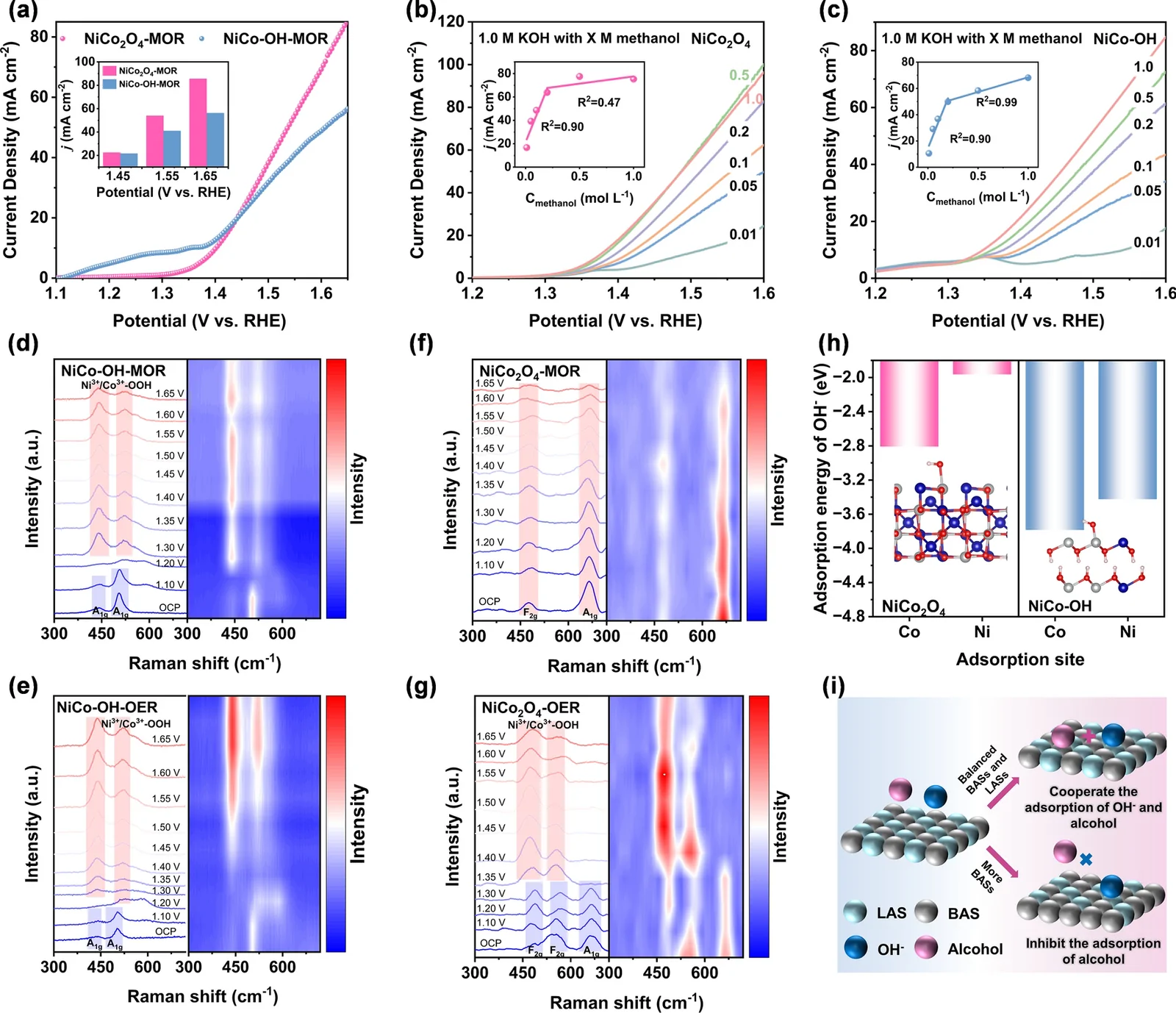
Performance analysis of methanol oxidation reaction (MOR) using NiCo–OH and NiCo_2_O_4_ solid-acid electrocatalysts. **a** LSV curves of NiCo–OH and NiCo_2_O_4_ by using 1 M KOH + 0.1 M methanol at a scan rate of 5 mV s^−1^ with the histograms of MOR current densities at different potentials in the inset. LSV curves of **b** NiCo–OH and **c** NiCo_2_O_4_ in 1.0 M KOH and different methanol concentrations (X = 0.01, 0.05, 0.1, 0.2, 0.5, and 1.0 M) at a scanning rate of 5 mV s^−1^ with the insets of corresponding relationships between MOR current density and methanol concentration at 1.55 V vs. RHE. In-situ Raman spectra of **d** MOR and **e** OER for NiCo–OH, and **f** MOR and **g** OER for NiCo_2_O_4_. **h** Adsorption energy of OH^−^ at Ni and Co sites of NiCo–OH and NiCo_2_O_4_. The catalyst models as shown with Ni, Co, O, and H atoms marked in gray, blue, red, and white, respectively. **i** Schematic diagram of a solid-acid electrocatalyst with balanced BASs and LASs promoting the reaction of OH^−^ and alcohols molecules, and adsorption of alcohol molecules could be inhibited on that with more BASs

The effect of acid sites of NiCo–OH and NiCo_2_O_4_ on the activity of OER and methanol oxidation reaction (MOR) was firstly investigated by performing the electrochemical experiments in 1 M KOH with or without 0.1 M methanol electrolyte. The linear sweep voltammetry (LSV) curves for the MOR activity by NiCo–OH and NiCo_2_O_4_ are shown in Fig. 3a, and the currents at the overpotential of 1.45, 1.55, and 1.65 V vs. RHE are also compared (the inset in Fig. 3a). NiCo_2_O_4_ with balanced LASs and BASs can reach the current density of 22.5, 54.0, and 85.6 mA cm^−2^ during MOR. In contrast, NiCo–OH with more BASs only attains current density of 21.6, 41.0, and 56.2 mA cm^−2^. The bulge observed prior to 1.4 V vs. RHE can be ascribed to the oxidation peak of NiCo–OH, which is also evident from the cyclic voltammetry (CV) curves (Fig. S11). The charge transfer resistance of NiCo_2_O_4_ is also smaller than that of NiCo–OH, which further proves that NiCo–OH–derived spinel oxide NiCo_2_O_4_ is more favorable for MOR (Fig. S12). The OER performance of two solid-acid electrocatalysts was also compared. At 1.65 V vs. RHE, the current density for NiCo_2_O_4_ is 33.6 mA cm^−2^, evidently smaller than 49.1 mA cm^−2^ for NiCo–OH (Fig. S13). Notably, through LSV, CV, and electrochemical impedance spectroscopy (EIS), it was observed that NiCo–OH significantly enhances the OER activity due to the higher proportion of BASs (Figs. S13-S15). Therefore, the above results indicate that NiCo_2_O_4_ with balanced LASs and BASs exhibits superior activity compared to NiCo–OH under MOR conditions. However, NiCo–OH with more BASs demonstrates higher activity under OER conditions. These results imply a significant disparity in the mechanism between AOR and OER by solid-acid electrocatalysts.

In order to understand reaction mechanisms of NiCo-based electrocatalysts under MOR and OER conditions, we carried out a series of electrocatalytic experiments and density functional theory (DFT) calculations. As shown in Fig. 3b, c, the methanol concentration (*C*_methanol_) dependence for NiCo–OH and NiCo_2_O_4_ was studied at 1.0 M KOH with varying concentrations of methanol (X = 0.01, 0.05, 0.1, 0.2, 0.5, and 1.0 M). When *C*_methanol_ is between 0.01 and 0.2 mol L^−1^, the relationship between *C*_methanol_ and current density is linear, indicating that MOR is primarily a diffusion-controlled process at low *C*_methanol_ [47]. When *C*_methanol_ > 0.2 mol L^−1^, the current density still increases linearly, but the slope at NiCo_2_O_4_ is small, mainly because the kinetic control factors other than diffusion control factors may affect the increase in current density. For NiCo_2_O_4_, the current density decreases at higher *C*_methanol_ because methanol has a stronger adsorption capacity on NiCo_2_O_4_ (Fig. 3b), depriving the locally adsorbed OH* species [48–50]. Through Bode diagram analysis, the peak potential of NiCo_2_O_4_ is approximately 1.274 V, whereas NiCo–OH emerges at 1.324 V, indicating a superior performance of NiCo_2_O_4_ in MOR. With the potential increasing, the frequency of NiCo_2_O_4_ also exhibits a faster rise, implying efficient oxidation of adsorbed methanol and enhanced interface charge transfer (Fig. S16).

In order to further investigate the underlying factors contributing to the enhanced OER activity of NiCo–OH and the enhanced MOR activity of NiCo_2_O_4_, we conducted in-situ Raman analysis to characterize structural evolution during OER and MOR (Fig. 3d-g). For NiCo–OH, a distinct Raman peak associated with Ni^3+^–O or Co^3+^–O was observed at 1.3 V in KOH solutions containing methanol (Fig. 3d) and without methanol (Fig. 3e) [51]. This observation suggests that a higher proportion of BASs in NiCo–OH is more susceptible to oxidation (such as NiOOH or CoOOH), leading to the formation of active sites for OER. Consequently, this promotes OER while inhibiting MOR. In contrast, no characteristic peaks related to Ni^3+^–O or Co^3+^–O were detected during the MOR process with NiCo_2_O_4_ (Fig. 3f). This finding indicates that balanced LASs and BASs facilitate faster MOR on NiCo_2_O_4_ while effectively maintaining stable valence states for both Ni and Co. It is worth noting that during OER, Raman peaks associated with Ni^3+^–O or Co^3+^–O gradually emerge only when reaching a potential of 1.35 V. It indicates that OER was hampered on NiCo_2_O_4_ (Fig. 3g).

The high MOR activity of NiCo_2_O_4_ is further supported by theoretical calculation. As shown in Figs. 3h and **S17-S18**, the adsorption energy and differential charge density of OH^−^ on the surfaces of NiCo_2_O_4_ and NiCo–OH were calculated using DFT. A periodic surface model based on NiCo_2_O_4_ and NiCo–OH was established, as shown in Fig. S17, along with the adsorption of OH^−^ on the surface. A significantly lower adsorption energy of OH^−^ at the Co site compared to the Ni site suggests the Co site was the main active site for the adsorption of OH^−^ (Fig. 3h). And the adsorption energy of OH^−^ at the Co site of NiCo–OH (− 3.79 eV) exhibited significantly lower value compared to that observed at the Co site of NiCo_2_O_4_ (− 2.81 eV). The differential charge analysis reveals electron transfer between Co and the adsorbent OH^−^, following a ‘receive-donate’ mechanism (Fig. S18). Bader charge analysis shows that the electron transfer between OH^−^ and NiCo_2_O_4_ was 0.99 e, and the electron transfer number between OH^−^ and NiCo–OH was 1.07 e. Therefore, it is proved that NiCo–OH has stronger interaction with OH^−^ and can promote OER [52, 53]. Strong adsorption leads to the inability of methanol and OH^−^ to co-adsorb on NiCo–OH surfaces, thus inhibiting MOR [54]. These results indeed confirm that NiCo_2_O_4_ with balanced LASs and BASs has the most suitable OH* and methanol adsorption energy, which can cooperate with the adsorption of OH* and methanol. In sharp contrast, NiCo–OH has more BASs and less LASs, leading to strong adsorption of OH* and inhibiting adsorption of methanol molecules (Fig. 3i).

### Electrocatalytic Oxidations of C_1_-C_6_ Saturated Alcohols by NiCo_2_O_4_ Solid-Acid Electrocatalysts with Balanced LASs and BASs

Additionally, we accessed the performance of both NiCo–OH and NiCo_2_O_4_ toward sorbitol oxidation reaction (SOR, details in Figs. S19 and S20). These findings not only demonstrate the superior SOR performance for NiCo_2_O_4_, but also suggest higher activity for polyhydroxy-based SOR compared to MOR. Notably, the same results were obtained for C_2_-C_5_ alcohols, both of which showed excellent alcohol oxidation activity of NiCo_2_O_4_ (Fig. S21). The product test results of NiCo–OH and materials of different proportions of LASs/BASs indicated that NiCo_2_O_4_ containing balanced proportion of LASs and BASs is the best-performing AOR electrocatalyst (Figs. S22-S24 and Table S4).

**Fig. 4:**
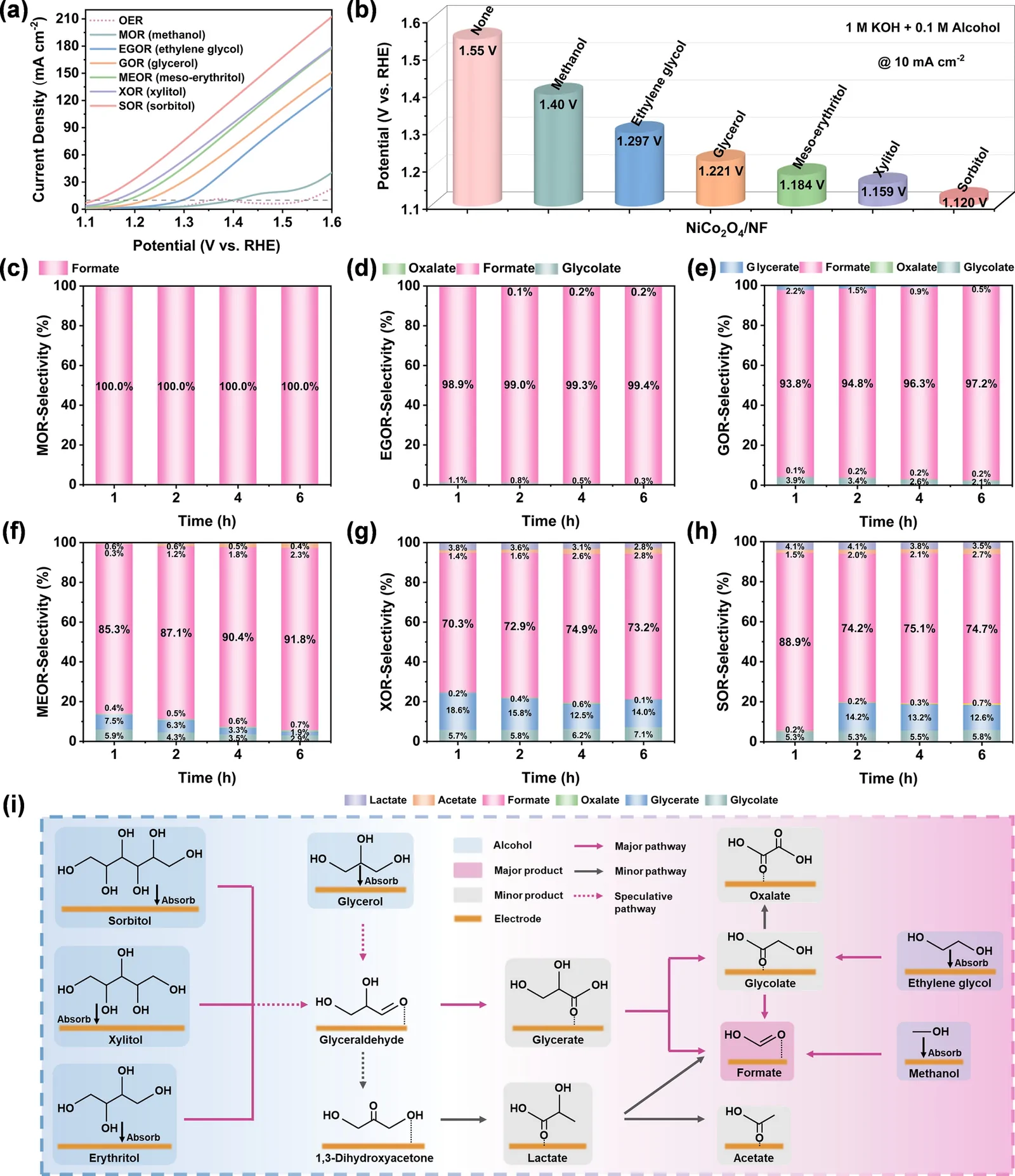
Electrocatalytic oxidations performances of C_1_-C_6_ saturated alcohols on NiCo_2_O_4_ with balanced BASs and LASs. **a** LSV curves (without iR-correction) of NiCo_2_O_4_ in 1 M KOH or 1 M KOH with 0.1 M C_1_-C_6_ saturated alcohol (e.g., methanol, ethylene glycol, glycerol, meso-erythritol, xylitol, and sorbitol) at a scan rate of 5 mV s^−1^. **b** Histograms of the potentials required to achieve a current density of 10 mA cm^−2^ for the oxidation of C_1_-C_6_ saturated alcohols on NiCo_2_O_4_ electrodes. Product selectivity for the oxidation of **c** methanol, **d** ethylene glycol, **e** glycerol, **f** meso-erythritol, **g** xylitol, and **h** sorbitol. **i** Proposed reaction pathways for the electrochemical oxidation of C_1_-C_6_ saturated alcohols on NiCo_2_O_4_ solid-acid electrocatalyst with balanced BASs and LASs

Furthermore, we compared the oxidation ability of various saturated alcohols (e.g., methanol, ethylene glycol, glycerol, meso-erythritol, xylitol, and sorbitol) with increased carbon chain lengths (C_1_-C_6_) using NiCo_2_O_4_ with balanced LASs and BASs. Initially, we conducted LSV analysis in a three-electrode system using 1 M KOH with 0.1 M alcohol solutions. As depicted in Fig. 4a, minimal reaction was occurred in the absence of alcohol. However, upon the addition of saturated alcohol molecules, the current density improved progressively with increasing hydroxyl group contents in alcohol molecules. The overpotential at 10 mA cm^−2^ is depicted in Fig. 4b, illustrating a clear enhancement in oxidation activity with higher hydroxyl group contents: OER (1.550 V) < methanol (1.400 V) < ethylene glycol (1.297 V) < glycerol (1.221 V) < meso-erythritol (1.184 V) < xylitol (1.159 V) < sorbitol (1.120 V). Furthermore, when the number of hydroxyl groups of saturated molecules in the control solution is the same (as shown in Fig. S25), it can be observed that with the increase in the number of hydroxyl groups in saturated alcohol molecules, their activity also shows a gradually increasing trend. These findings indicate that the number of hydroxyl groups indeed influences alcohol oxidation activity, with more hydroxyl groups correlating to easier oxidation of alcohols.

In addition, quantitative analysis of products is essential for gaining deep insights into the mechanisms of alcohol oxidation by NiCo_2_O_4_ solid-acid electrocatalysts. High-performance liquid chromatography (HPLC) was employed to characterize the products resulting from the electrocatalytic oxidation of six saturated alcohols by NiCo_2_O_4_ (Figs. S26 and S27). Comparative analysis with standard samples reveals that formate was identified as the primary product for all AORs, accompanied by secondary products including acetate, lactate, oxalate, glycerate, and glycolate. This detailed product analysis provides valuable insights into the reaction pathways involved in alcohol oxidation catalyzed by NiCo_2_O_4_, contributing to a comprehensive understanding of electrocatalytic processes in alcohol oxidation within the realm of energy-related research. As illustrated in Fig. 4c, during the MOR process, the simplicity of its products, primarily formate, is attributed to its status as a monohydric saturated alcohol, resulting in a remarkable selectivity of 100%. Conversely, with an increase in the length of the carbon chain, the emergence of minor by-products becomes apparent. For instance, in the reaction of ethylene glycol oxidation (EGOR) observed at 2 h (Fig. 4d), a small amount of glycolate (0.8%) and oxalate (0.1%) were detected. Furthermore, as the reaction progressed, the concentration of the primary product, formate, exhibited a gradual increase and ultimately achieved a remarkable selectivity of 99.4% the resulting products encompass formate, glycerate, glycolate, and oxalate. As the duration of the reaction increases, there is a corresponding elevation in product concentrations, with peak formate production rate observed. The formate selectivity increases from the initial 93.8% to 97.2% (Fig. 4e). Upon oxidation of meso-erythritol (MEOR) (Fig. 4f), due to the presence of four carbon–carbon bonds, the range of by-products expands beyond formate to include acetate, lactate, oxalate, glycerate, and glycolate. Consequently, the selectivity for formate exhibits a slight decrease, reaching 91.8% after 6 h reaction. Similarly, in the oxidation of pentitol (xylitol, XOR) and hexitol (sorbitol, SOR), although the carbon chain length increases, the primary products remain consistent with C_1_-products (formate), C_2_-products (acetate, glycolate and oxalate), and C_3_-products (glyceate and lactate) (Fig. 4g, h). Similarly, the comparison of product selectivity at different potentials also shows consistency (Figs. S28-S30). The findings demonstrate that the synergistic LASs and BASs in NiCo_2_O_4_ exhibit high formate selectivity and electrocatalytic activity across C_1_-C_6_ saturated alcohols. However, an increase in carbon chain length results in the production of more by-products, thus slightly decreasing the formate selectivity.

Based on the results presented, we have proposed potential reaction pathways for the electrocatalytic oxidation of six saturated alcohols using NiCo_2_O_4_ with balanced BASs and LASs, as illustrated in Fig. 4i. Red arrows denote inferred primary pathways, while black arrows indicate potential secondary pathways involved in the electrochemical processes. For MOR and EGOR, which exhibit lower reactivity, the primary pathway involves oxidation to formate with exceptional selectivity ranging from 99% to 100%. In the case of EGOR (a two-carbon alcohol), carbon–carbon bond cleavage results in the formation of small amounts of glycolate, followed by further oxidation leading to minor oxalate or eventual conversion into formate. The primary pathway for GOR initiates with the conversion of glycerol to glycerate, followed by C–C bond oxidation resulting in glycolate and formate. Similar to EGOR, the formed glycolate undergoes subsequent oxidation to generate oxalate or convert into formate. For alcohols with more than three carbons (meso-erythritol, xylitol, and sorbitol), the complexity of product profiles increases due to the presence of additional carbon–carbon bonds. The primary pathway involves initial oxidation to glyceraldehyde, followed by glycerate formation, and subsequent C–C oxidation leading to glycolate and formate. Further oxidation results in oxalate or ultimately formate. A secondary pathway involves the oxidation of glyceraldehyde to 1,3-dihydroxyacetone, followed by further oxidation to lactate, and finally, lactate oxidation to acetate or splitting into formate. These proposed reaction pathways offer valuable insights into the complex transformation of saturated alcohols under NiCo_2_O_4_ solid-acid electrocatalysts, emphasizing the significance of carbon chain length and bond cleavage in dictating product selectivity and overall electrocatalytic performance.

### Exploring Oxidation Mechanism of C_1_-C_6_ Saturated Alcohols on NiCo_*2*_O_4_ with Balanced LASs and BASs

**Fig. 5:**
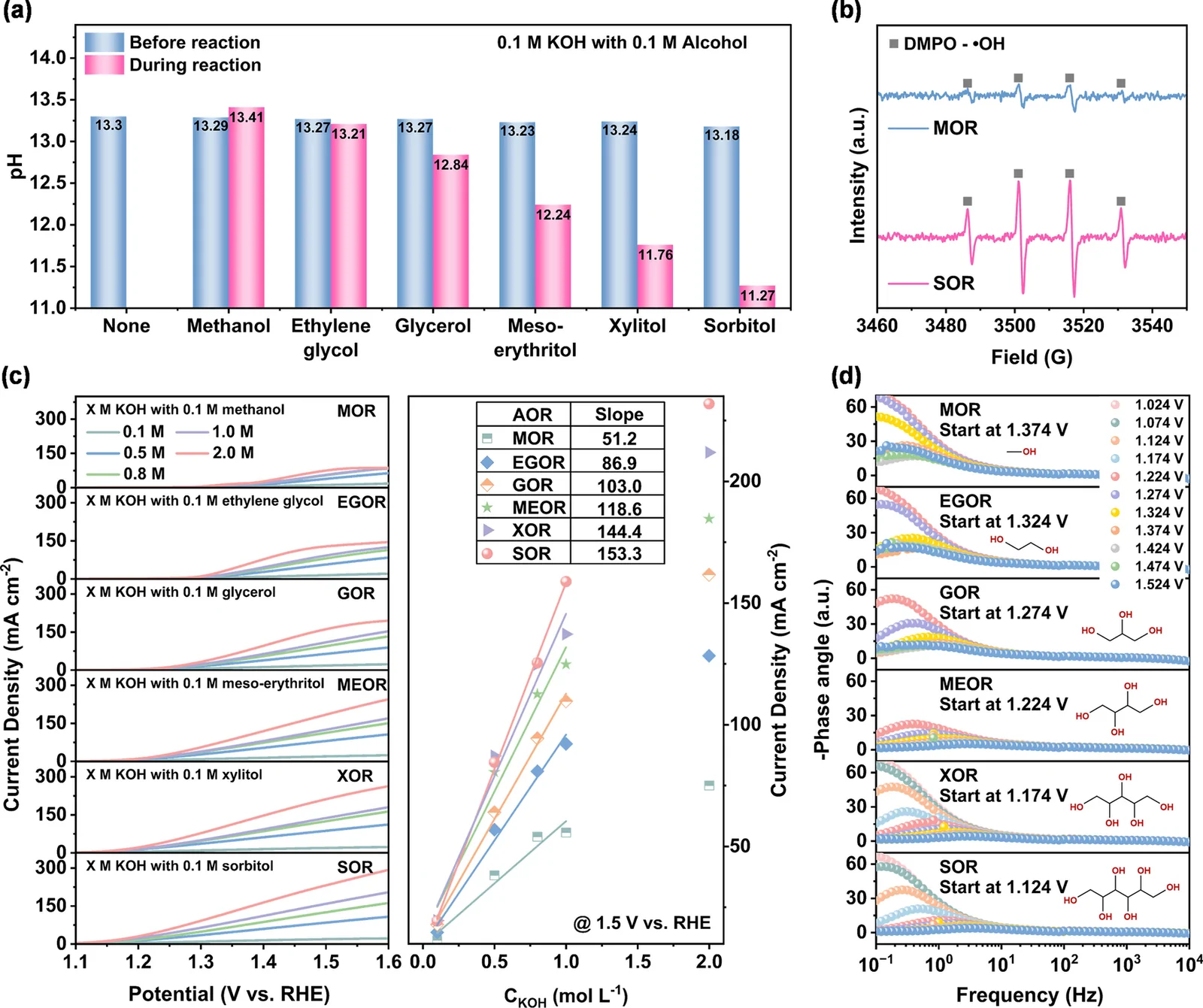
Reaction mechanism and charge transfer kinetics of the oxidation of C_1_-C_6_ saturated alcohols on NiCo_2_O_4_ surface. **a** Histograms of the pH values at near surface of NiCo_2_O_4_ before and during the reaction in 0.1 M KOH with 0.1 M C_1_-C_6_ saturated alcohols. **b** ESR detection of hydroxyl radicals in electrolytes using DMPO as a trapping agent in MOR and SOR catalyzed by NiCo_2_O_4_. **c** Left: LSV curves of NiCo_2_O_4_ at different KOH concentrations (X = 0.1, 0.2, 0.5, 0.8, 1.0, and 2.0 M) with 0.1 M C_1_-C_6_ saturated alcohols at a scanning rate of 5 mV s^−1^. Right: Relationship between AOR current density and KOH concentration at 1.5 V vs. RHE. **d** Bode diagrams of various AOR in 1.0 M KOH with 0.1 M C_1_-C_6_ saturated alcohol catalyzed by NiCo_2_O_4_ at different potentials

In order to elucidate the underlying mechanism behind the observed enhancement in electrocatalytic activity with an increasing number of hydroxyl groups in saturated alcohols, a series of experiments were also conducted. The pH variation near the catalyst surface during the electrocatalytic reaction in 0.1 M KOH with 0.1 M C_1_-C_6_ saturated alcohol was detected. The pH of the electrolyte itself is shown in the blue column in Fig. 5a. The pH is about 13.2 with or without alcohol molecules. When AOR occurs, the near-surface pH value decreases with the increase of the number of hydroxyl groups in the saturated alcohol itself. This may be due to the fact that more OH^−^ is consumed leading to a decrease in near-surface pH. Specifically, a higher number of saturated alcohol hydroxyl groups requires more OH* to participate in the reaction, thus increasing catalytic activity. The electron spin resonance (ESR) test confirmed this finding (Fig. 5b), which were further supported by a higher concentration of hydroxyl radicals observed in the electrolyte following SOR compared to MOR [55].

The pH dependence measurements of NiCo_2_O_4_ were performed at X M (X = 0.1, 0.5, 0.8, 1.0, and 2.0) KOH with 0.1 M alcohol, as shown in Fig. 5c. The left panel of Fig. 5c represents the LSV curves of NiCo_2_O_4_ in the solution of different OH^−^ concentrations (*C*_KOH_) and 0.1 M alcohol molecules. The dependence between the AOR current density at 1.5 V and the *C*_KOH_ was calculated to derive the corresponding data presented in the right panel of Fig. 5c. The AOR current density of NiCo_2_O_4_ exhibits a consistent linear increase within the *C*_KOH_ = 0.1–1.0 M. However, when *C*_KOH_ > 1.0 M, the change in current density is no longer linear. This phenomenon can be attributed to the competitive adsorption between alcohol molecules and OH* species. The high concentration of OH^−^ occupies more active sites, hindering the adsorption of small molecules and further reducing AOR current density. In all AORs, the correlation between current density and KOH concentration exhibits an increasing slope with higher hydroxyl numbers of saturated alcohols, suggesting a strengthening degree of proton-electron decoupling transfer [47].

Figure 5d shows the Bode plots of NiCo_2_O_4_ during AOR. It can be observed that a significant transition peak emerges in the low-frequency region at an increased potential of 1.375 V, indicating the initiation of MOR at this potential. It can be seen that when MOR reaction occurs with the potential increasing to 1.375 V, there is an obvious transition peak in the low-frequency region (0.1–10 Hz), indicating that MOR begins at a potential of ~ 1.375 V. This suggests that hydroxyl groups may also be involved in the AOR process [56]. The potential of the characteristic peak decreases with an increase in the number of hydroxyl groups during AOR. The order of decreasing potential is as follows: MOR (~ 1.375 V) < EGOR (~ 1.324 V) < GOR (~ 1.274 V) < MEOR (~ 1.224 V) < XOR (~ 1.174 V) < SOR (~ 1.124 V). The peak is related to the uneven charge contribution generated by the reaction between OH* and alcohols. As the potential increases, it slowly shifts toward higher frequencies and lower angles. This means that absorbed alcohols are rapidly oxidized, resulting in faster interfacial charge transfer. In other words, the more hydroxyl groups saturated alcohol molecules have, the faster the interfacial charge transfer occurs, which is more favorable for the reaction to take place [47].

### Revealing Oxidation Mechanism of C_1_-C_6_ Saturated Alcohols by DFT Calculations

**Fig. 6:**
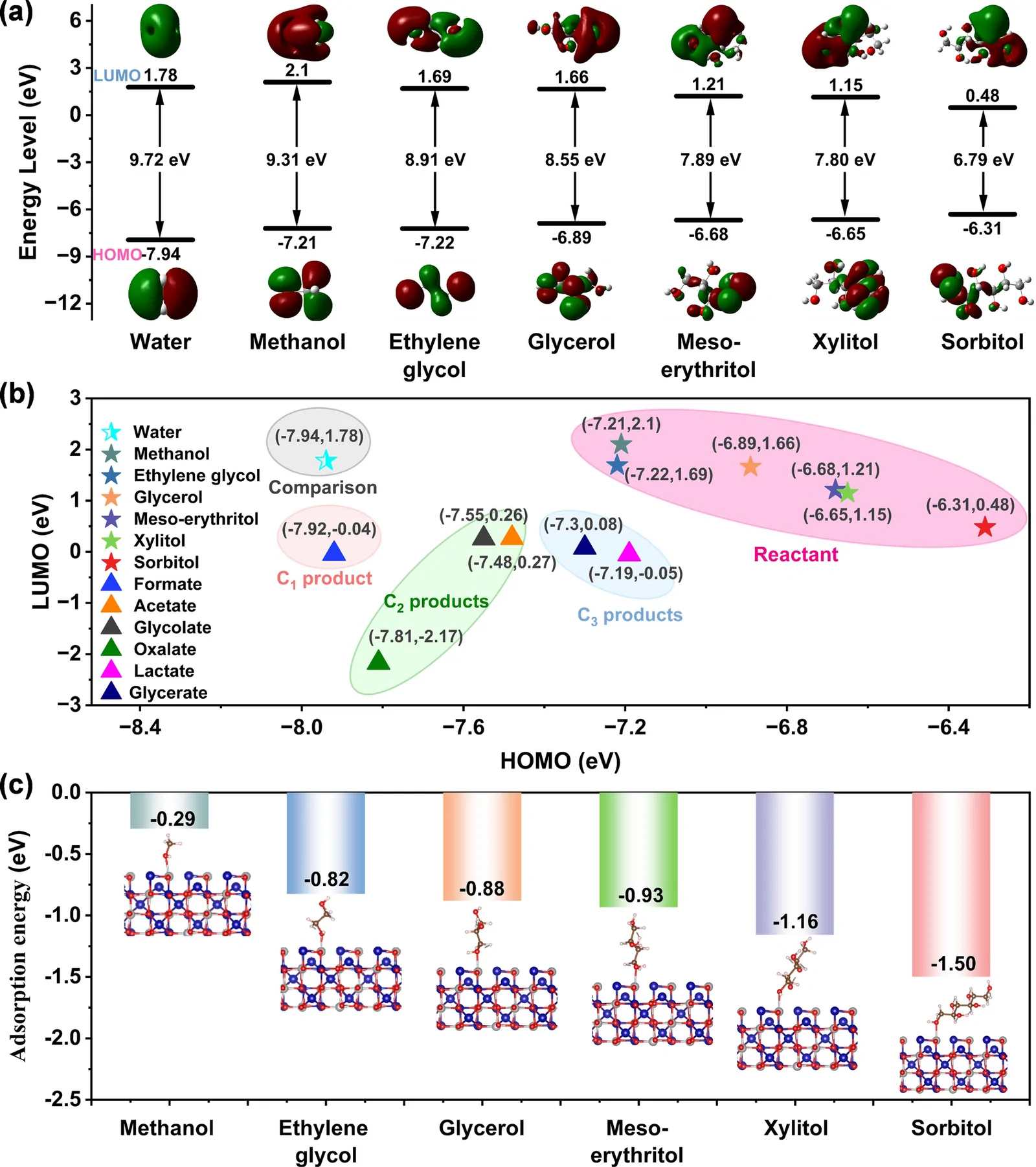
DFT analysis. **a** HOMO orbitals, LUMO orbitals and HOMO–LUMO gap of water and C_1_-C_6_ saturated alcohols. **b** Comparison of HOMO and LUMO orbitals of water, C_1_-C_6_ saturated alcohols, C_1_-product (formate), C_2_-products (acetate, glycolate, oxalate), and C_3_-products (lactate, glycerate). **c** Adsorption energy of C_1_-C_6_ saturated alcohols on NiCo_2_O_4_ surface. The insets in **c** show the corresponding structural models of alcohol molecules adsorbed on NiCo_2_O_4_. The primary hydroxyl groups of alcohol molecules are adsorbed on the NiCo_2_O_4_ (110) surface

In addition, density functional theory (DFT) calculation was performed to study electron distribution of alcohol molecules. Theoretically, the highest occupied molecular orbital (HOMO) and lowest unoccupied molecular orbital (LUMO) values reflect the oxidation and reduction trends of molecules. The smaller the energy gap between HOMO and LUMO, the less chemically stable they are. The structural models of C_1_-C_6_ saturated alcohols are shown in Fig. S31. As shown in Fig. 6a, with the increase of the number of hydroxyl groups in saturated alcohols, the HOMO–LUMO gap value of alcohol molecules gradually decreases and is lower than that of H_2_O (9.72 eV): H_2_O (9.72 eV) < methanol (9.31 eV) < ethylene glycol (8.91 eV) < glycerol (8.55 eV) < meso-erythritol (7.89 eV) < xylitol (7.80 eV) < sorbitol (6.79 eV). Moreover, the ability to lose electrons can be further characterized at the molecular orbital level, as shown in Fig. 6a [57, 58]. The energies of the HOMO of alcohol molecules are as follows: methanol (− 7.21 eV) > ethylene glycol (− 7.22 eV) > glycerol (− 6.89 eV) > meso-erythritol (− 6.68 eV) > xylitol (− 6.65 eV) > sorbitol (− 6.31 eV), and all are higher than that of H_2_O (− 7.9 eV). Therefore, it can be inferred that in the adsorption process, electrons are more likely to transfer from alcohol molecules to the surface of NiCo_2_O_4_. And the more hydroxyl groups in the saturated alcohol molecules, the stronger their electron transfer ability and the easier to react at the electrode/electrolyte interface, which is consistent with the experimental results.

Additionally, as shown in Figs. S32 and S33, the HOMO and LUMO energies of the products measured by HPLC (C_1_-products: formate, C_2_-products: acetate, glycolate and oxalate; and C_3_-products: glycerate and lactate) were also calculated. These results are further summarized alongside the HOMO and LUMO energies of saturated alcohols, as depicted in Fig. 6b. It can be found that the saturated alcohols in the rose range are more easily oxidized in the upper right corner. Formate predominates as the primary product of alcohol oxidation due to its possession of the lowest HOMO and LUMO energy levels, consequently resulting in an elevated electron transfer barrier for molecular adsorption and conversion. This observation also elucidates why alcohol molecules are more readily oxidized compared to H_2_O molecules; formate serves as the prevailing product of most alcohol oxidation reactions by solid-acid electrocatalysts.

Finally, the adsorption Gibbs free energy of saturated alcohol molecules on the surface of NiCo_2_O_4_ was determined using DFT calculations, as depicted in Fig. 6c. The periodical surface models were built up based on NiCo_2_O_4_ with different alcohol molecules as shown in Fig. S34 and the insets of Fig. 6c**.** Notably, our results revealed a positive correlation between the number of hydroxyl groups and the adsorption energy on the electrocatalyst surface: Methanol (− 0.29 eV) < ethylene glycol (− 0.82 eV) < glycerol (− 0.88 eV) < meso-erythritol (− 0.93 eV) < xylitol (− 1.16 eV) < sorbitol (− 1.50 eV). This observation suggests that an increased number of hydroxyl groups leads to enhanced activity in the AOR reaction.

## Conclusions

In summary, we studied how BASs and LASs in NiCo-based solid-acid electrocatalysts impact the oxidation activity of a series of C_1_-C_6_ saturated alcohols by combining computational and experimental approaches. Layered double hydroxide NiCo–OH with a higher proportion of BASs (89.6%) facilitates the metal site oxidation to high-valence state OOH^III^ species for enhanced OER activity. After heat treatment, NiCo–OH–derived NiCo_2_O_4_ with balanced BASs (46.9%) and LASs (53.1%) facilitates co-adsorption of alcohols and OH^−^, thereby favoring the AOR and enabling highly selective formate production. Moreover, an intriguing trend was observed when oxidation of a series of C_1_ to C_6_ saturated alcohols. The increase of the number of hydroxyl groups corresponds to the elevated nucleophilicity and adsorption energy for alcohol molecules, leading to the significant activity AOR: methanol (C_1_) < ethylene glycol (C_2_) < glycerol (C_3_) < meso-erythritol (C_4_) < xylitol (C_5_) < sorbitol (C_6_). But the formate selectivity shows the opposite trend from 100 to 86%, accompany minor byproducts. This study provides deep insights of LASs and BASs affecting the OER and AOR, and also rationally guides design of solid-acid electrocatalysts with optimized proportions of acid sites for anodic oxidation processes. Meanwhile, the relationship between the number of hydroxyl groups in alcohol molecules and the enhancement of activity provides an important reference value for the design of non-noble metal catalysts and devices to reduce cell voltage in the future.

## Supplementary Information

Below is the link to the electronic supplementary material.

Supplementary file1 (DOCX 7547 KB)
