# Supplementary material for: Saturated Alcohols Electrocatalytic Oxidations on Ni-Co Bimetal Oxide Featuring Balanced B- and L-Acidic Active Sites
Source: Nanomicro Lett. 2025 Aug 25;18:39. doi: 10.1007/s40820-025-01893-z (PMC12378812; doi:10.1007/s40820-025-01893-z)
Supplement: Supplementary file 1 — Supplementary file1 (DOCX 7547 KB) [file 40820_2025_1893_MOESM1_ESM.docx]

Supplementary Information for

Saturated Alcohols Electrocatalytic Oxidations on Ni-Co Bimetal Oxide Featuring Balanced B- and L- Acidic Active Sites

Junqing Ma^1, 2, 3, #^, Wenshu Luo^1, 2, #^, Xunlu Wang^4, #^, Xu Yu^1, 2^, Jiacheng Jayden Wang^1, 2^, Huashuai Hu^4^, Hanxiao Du^1, 2^, Jianrong Zeng^5^, Wei Chen^6^, Minghui Yang^4^, Jiacheng Wang^3,^ *, and Xiangzhi Cui^1, 2, 7,^ *

^1^ State Key Laboratory of High-Performance Ceramics and Superfine Microstructure, Shanghai Institute of Ceramics, Chinese Academy of Sciences, Shanghai 200050, P. R. China

^2^ Center of Materials Science and Optoelectronics Engineering, University of Chinese Academy of Sciences, Beijing 100049, P. R. China

^3^ Zhejiang Key Laboratory for Island Green Energy and New Materials, Institute of Electrochemistry, School of Materials Science and Engineering, Taizhou University, Taizhou, Zhejiang 318000, P. R. China

^4^ School of Environmental Science and Technology, Dalian University of Technology, Dalian 116024, P. R. China

^5^ Shanghai Synchrotron Radiation Facility, Shanghai Advanced Research Institute, Chinese Academy of Sciences, Shanghai 201204, P. R. China

^6^ Department of Materials Design and Innovation, University at Buffalo, The State University of New York, Buffalo, NY 14260, USA

^7^ School of Chemistry and Materials Science, Hangzhou Institute for Advanced Study, University of Chinese Academy of Sciences, Hangzhou 310024, P. R. China

^#^Junqing Ma, Wenshu Luo and Xunlu Wang have contributed equally to this work.

*Corresponding authors. E-mail: [cuixz@mail.sic.ac.cn](mailto:cuixz@mail.sic.ac.cn) (Xiangzhi Cui); [jiacheng.wang@tzc.edu.cn](mailto:jiacheng.wang@tzc.edu.cn) (Jiacheng Wang)

**Supplementary Figures and Tables**
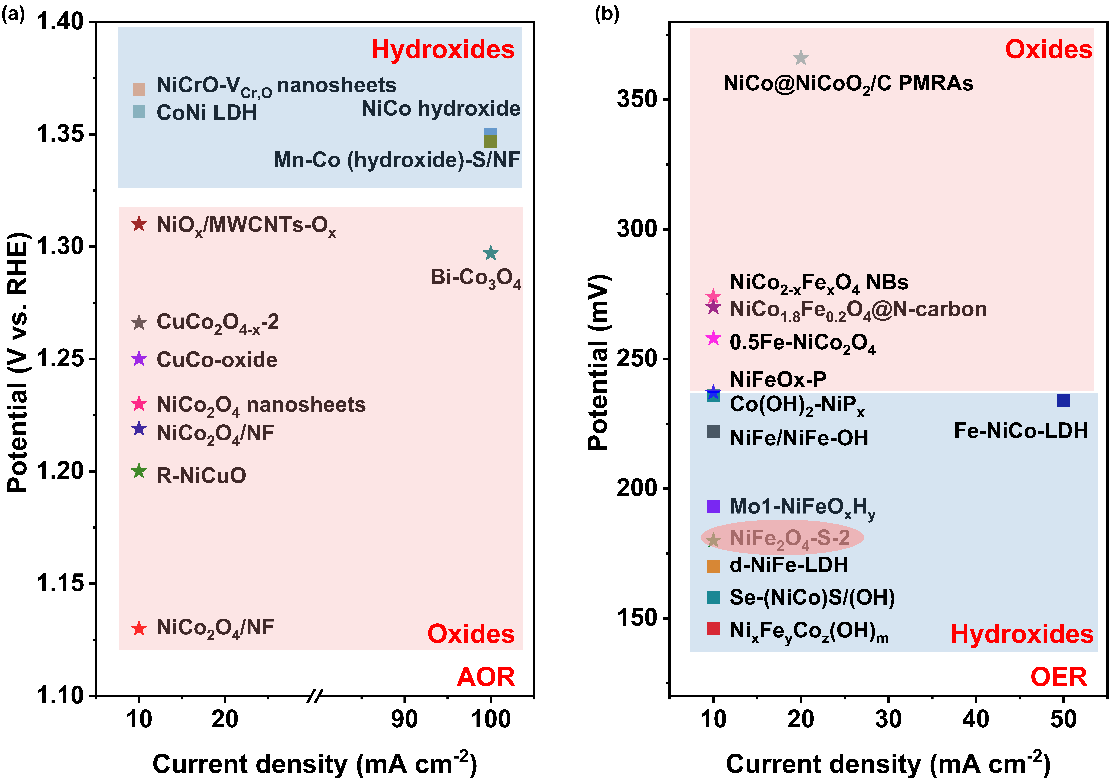


**Fig. S1** Comparison of (**a**) AOR and (**b**) OER activity of NiCo-based hydroxides and oxides electrocatalyst

Ni-based and Co-based catalysts are widely used transition metal electrocatalysts in the anodic oxidation reaction. After comprehensive summarization (Fig. S1 and Table S1-S2), it can be clearly observed that hydroxide have a wider distribution and higher activity in OER, while oxides generally exhibit good performance in AOR. However, there has been no detailed analysis or empirical research on this phenomenon by scholar. Therefore, this article first starts from the perspective of acid sites in NiCo-OH and NiCo_2_O_4_, comprehensively demonstrates and calculates their promotion effects on OER and AOR.


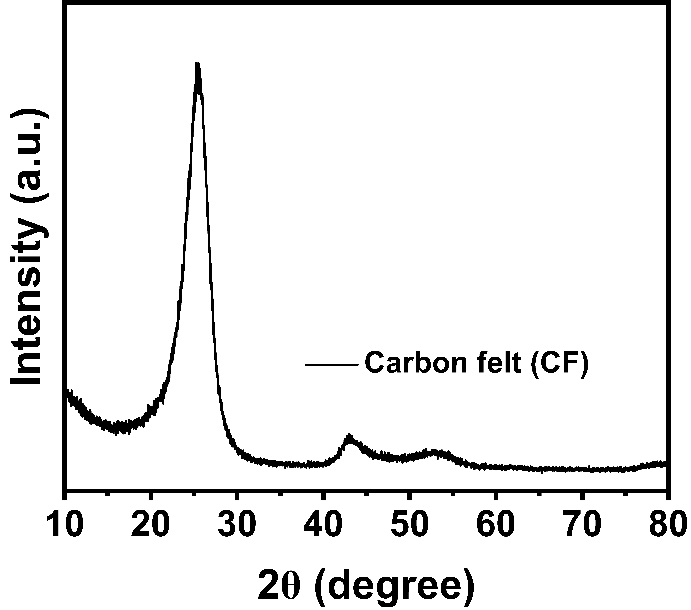


**Fig. S2** XRD pattern of carbon felt (CF)


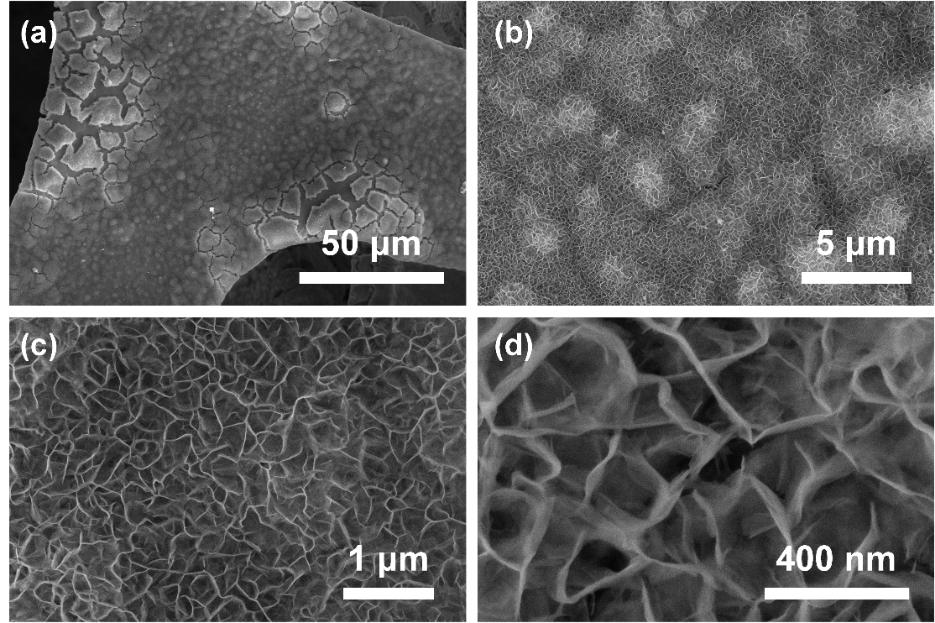


**Fig. S3** (**a-d**) Low and high-magnifications SEM images of NiCo-OH on NF


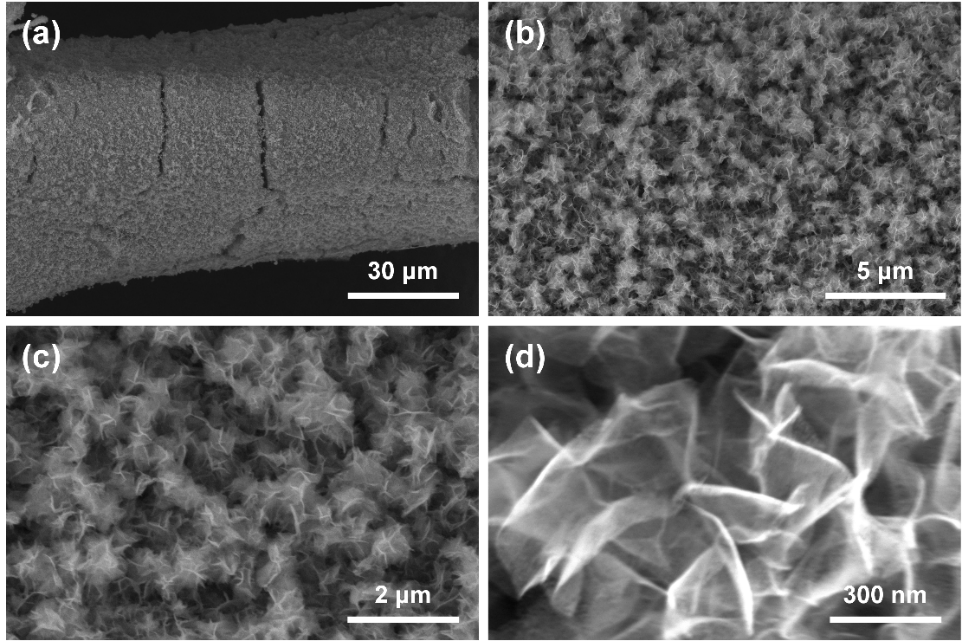


**Fig. S4** (**a-d**) Low and high-magnifications SEM images of NiCo_2_O_4_ on NF

**Fig. S5** TEM image of NiCo-OH on NF

**Fig. S6** TEM image of NiCo_2_O_4_ on NF


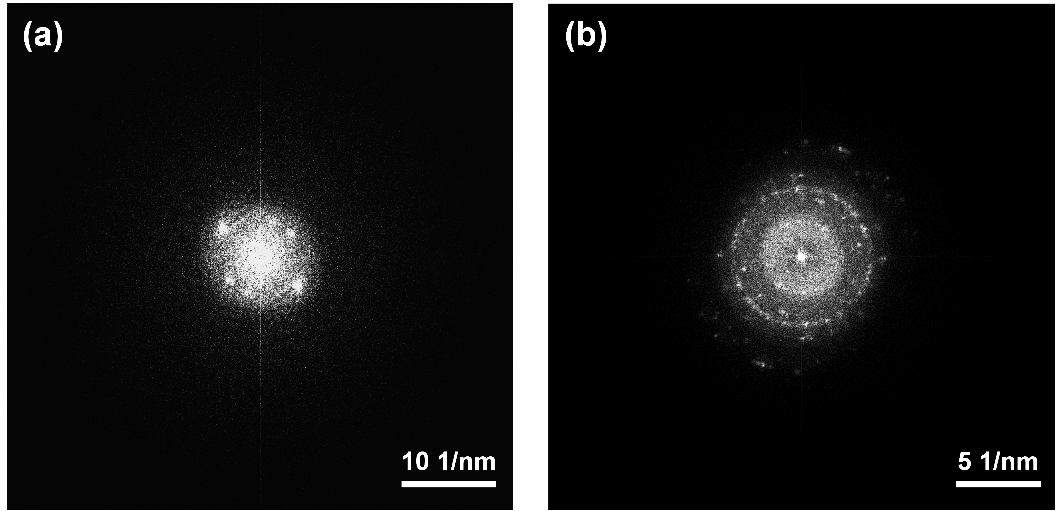


**Fig. S7** Fast Fourier transform (FFT) images of (**a**) NiCo-OH and (**b**) NiCo_2_O_4_


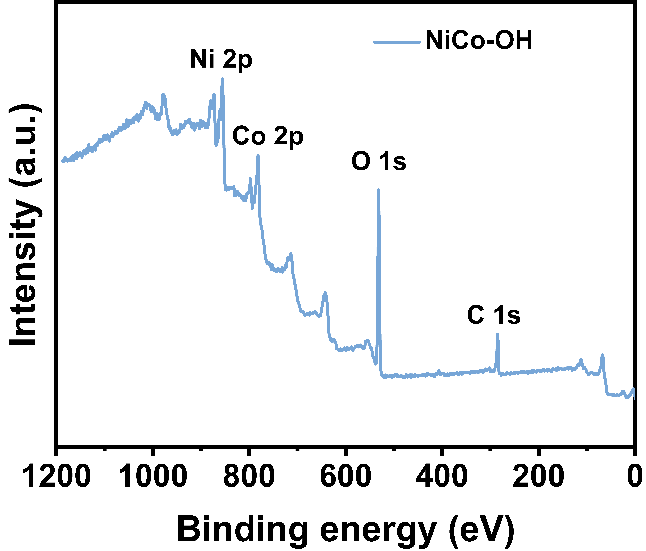


**Fig. S8** Overall XPS spectrum of NiCo-OH on NF


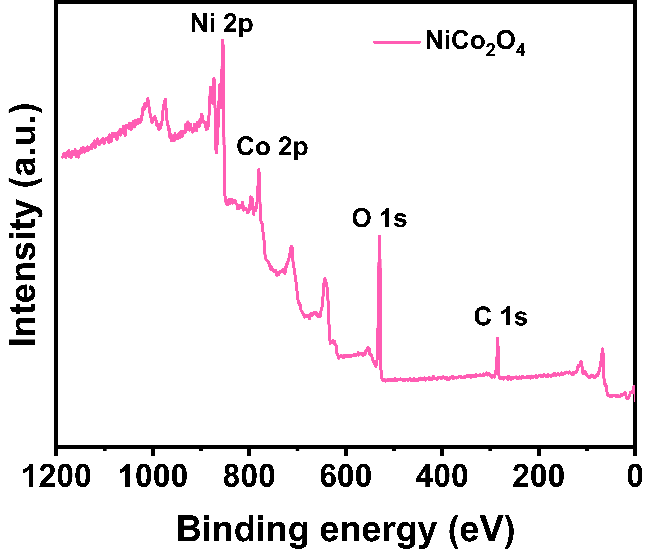


**Fig. S9** Overall XPS spectrum of NiCo_2_O_4_ on NF


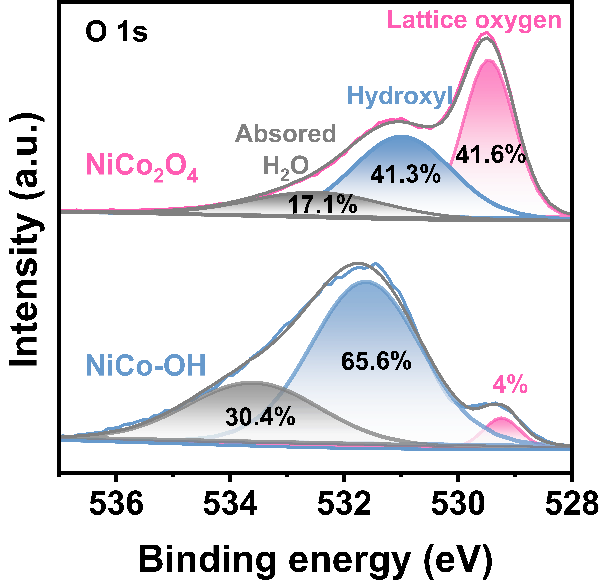


**Fig. S10** High-resolution XPS spectra for NiCo-OH and NiCo_2_O_4_ in the O 1s region


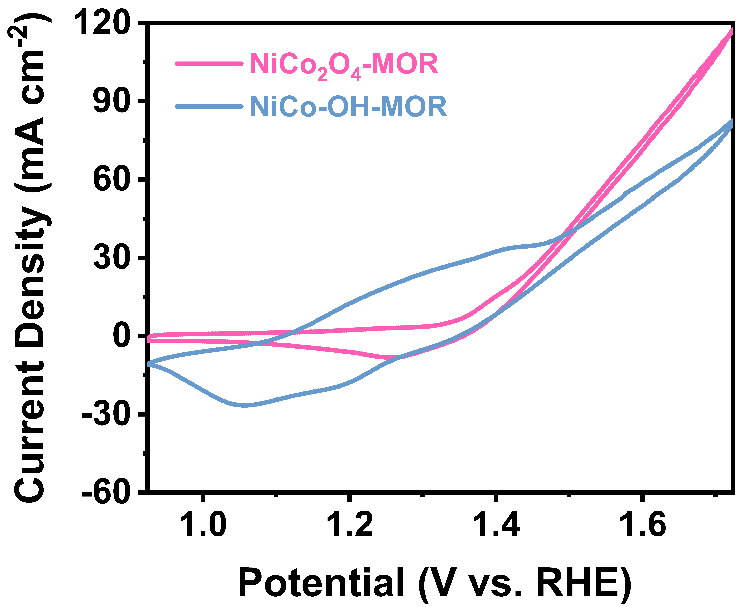


**Fig. S11** CV curves for NiCo-OH and NiCo_2_O_4_ in 1 M KOH with 0.1 M methanol at 100 mV s^-1^


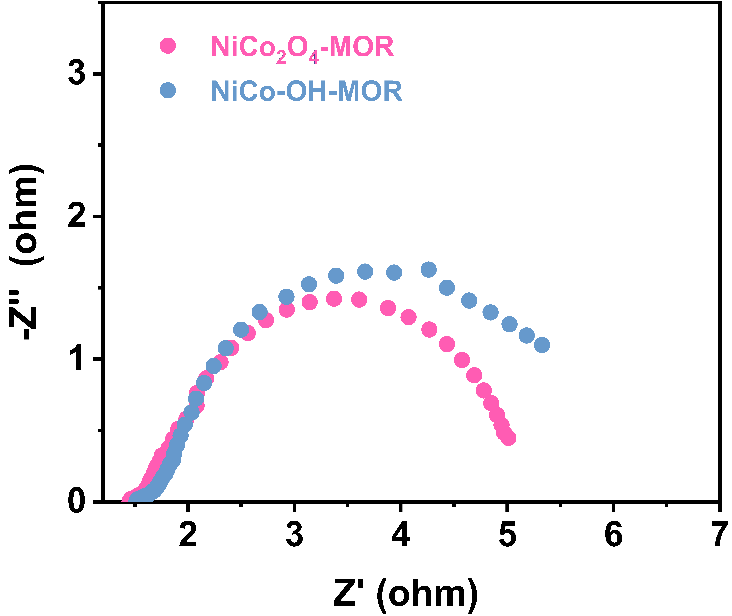


**Fig. S12** Electrochemical impedance spectroscopy of NiCo-OH and NiCo_2_O_4_ in 1 M KOH and 1 M KOH with 0.1 M methanol


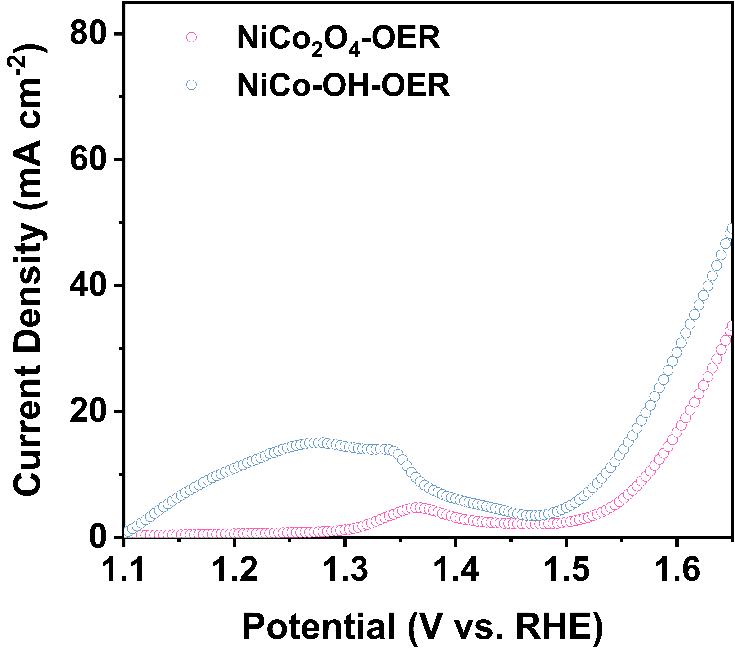


**Fig. S13** LSV curves of NiCo-OH and NiCo_2_O_4_ in 1 M KOH at a scan rate of 5 mV s^-1^


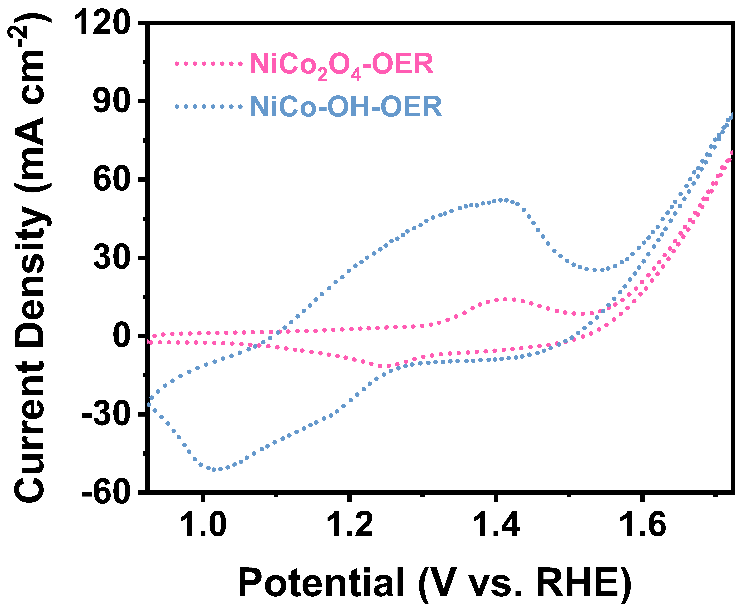


**Fig. S14** CV curves for NiCo-OH and NiCo_2_O_4_ in 1 M KOH at 100 mV s^-1^


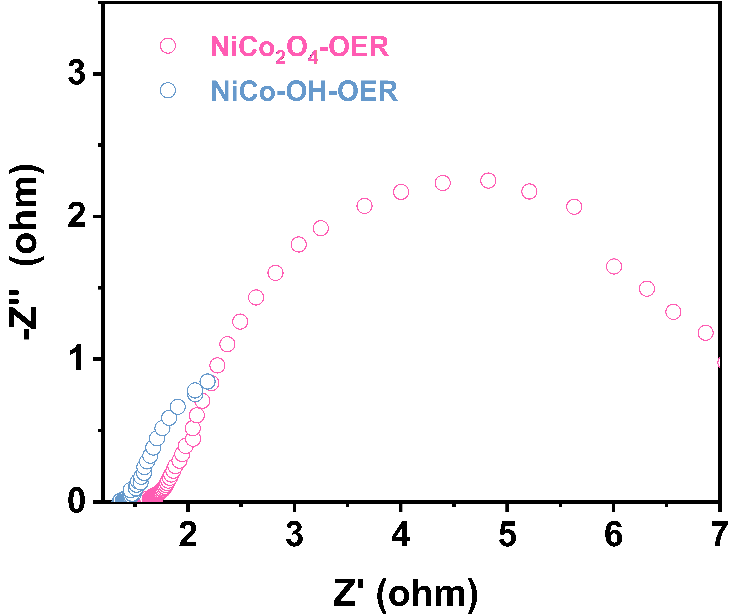


**Fig. S15** Electrochemical impedance spectroscopy of NiCo-OH and NiCo_2_O_4_ in 1 M KOH


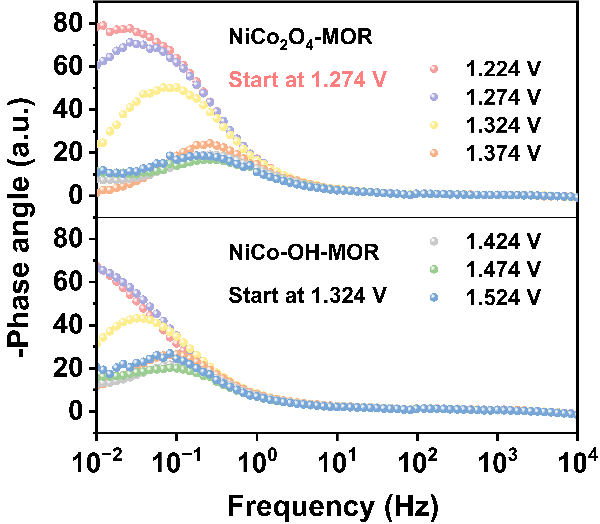


**Fig. S16** Bode diagram of NiCo-OH and NiCo_2_O_4_ in 1.0 M KOH with 0.1 M methanol


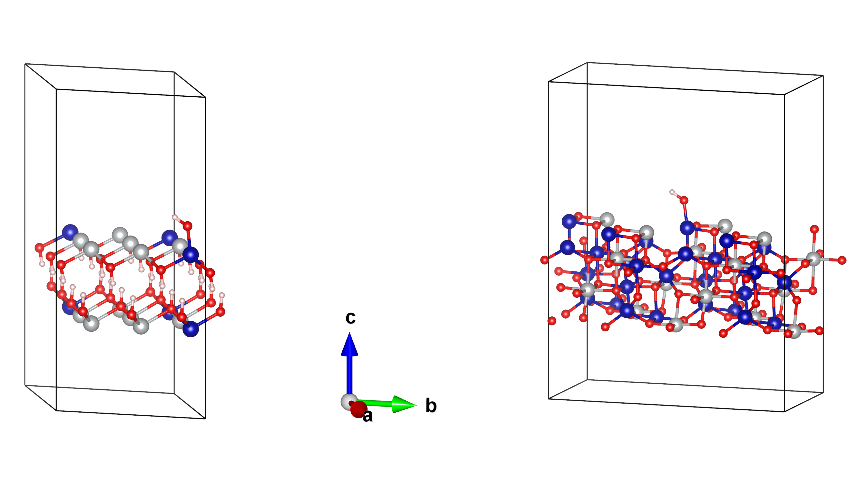


**Fig. S17** Model diagram of OH^-^ adsorbed at the Co sites on the surfaces of NiCo-OH and NiCo_2_O_4_


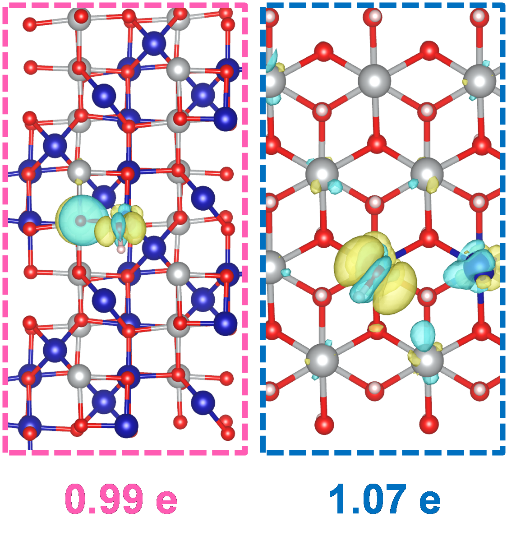


**Fig. S18** Differential charge density maps of OH^-^ at the Co sites of NiCo-OH and NiCo_2_O_4_


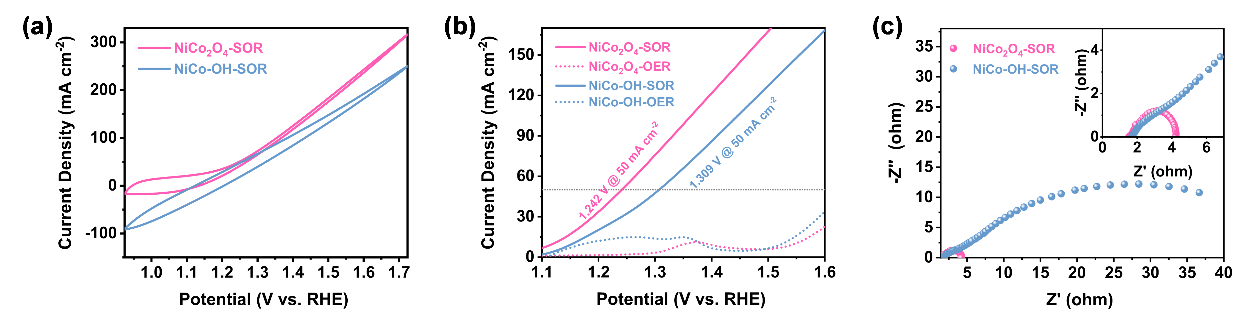


**Fig. S19** (**a**) CV curves for NiCo-OH and NiCo_2_O_4_ in 1 M KOH with 0.1 M sorbitol at 100 mV s^-1^. (**b**) SOR and OER polarization curves at 5 mV s^-1^ and (**c**) electrochemical impedance spectroscopy in 1 M KOH with 0.1 M sorbitol at 1.174 V vs. RHE of NiCo-OH and NiCo_2_O_4_


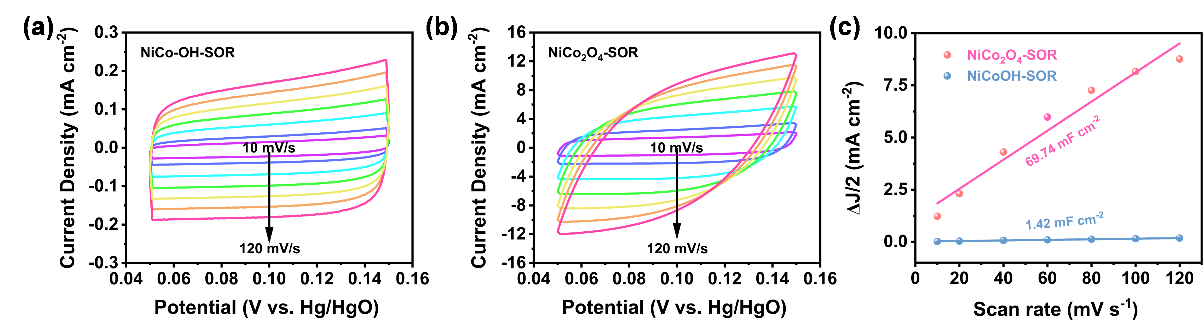


**Fig. S20** Cyclic voltammograms curves of (**a**) NiCo-OH and (**b**) NiCo_2_O_4_ in 1 M KOH with 0.1 M sorbitol at different scan rates. (**c**) The extracted double-layer capacitances (C_dl_) of NiCo-OH and NiCo_2_O_4_

The CV curves reveal that NiCo_2_O_4_ achieves a lower potential relative to NiCo-OH (Fig. S19a). Furthermore, LSV reveals that the sorbitol oxidation reaction (SOR) exhibits an onset potential of only 1.242 V vs. RHE at 50 mA cm^-2^ for NiCo_2_O_4_, while NiCo-OH demonstrates a larger onset potential of 1.309 V vs. RHE (Fig. S19b). Moreover, EIS confirms the lower charge transfer resistance of NiCo_2_O_4_, enhancing electron transfer kinetics for SOR (Fig. S19c). And NiCo_2_O_4_ has a higher double-layer capacitance (C_dl_) value (69.74 mF cm^-2^) than NiCo-OH (1.42 mF cm^-2^), suggesting that NiCo_2_O_4_ has a much larger electrochemical surface area and more active sites, which is conductive to the improvement of SOR activity (Fig. S20). These findings not only demonstrate the superior xylitol oxidation performance for NiCo_2_O_4_, but also suggest a higher activity for polyhydroxyl-based SOR compared to MOR.


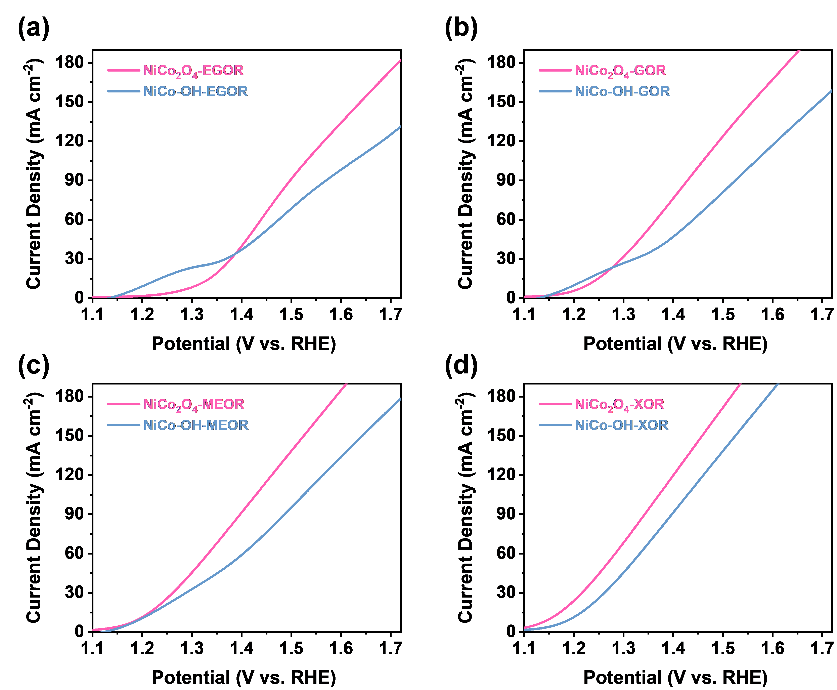


**Fig. S21** LSV curves of NiCo-OH and NiCo_2_O_4_ by using 1 M KOH with 0.1 M (**a**) ethylene glycol, (**b**) glycerol, (**c**) meso-erythritol and (**d**) xylitol at a scan rate of 5 mV s^-1^


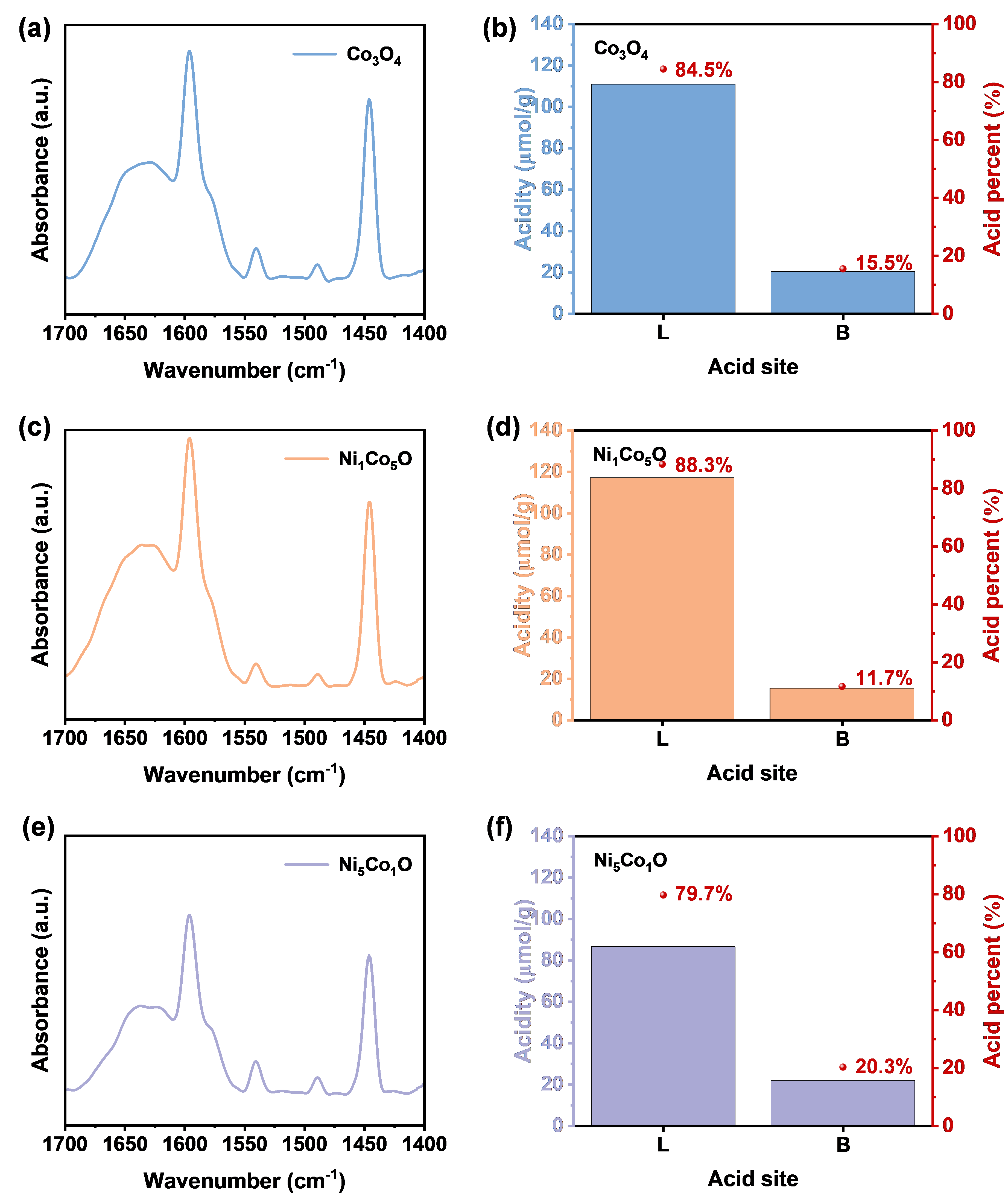


**Fig. S22** (**a**) FTIR spectra of Co_3_O_4_ after pyridine adsorption. (**b**) Histograms of acidity and corresponding ratios of LASs and BASs in Co_3_O_4_. (**c**) FTIR spectra of Ni_1_Co_5_O after pyridine adsorption. (**d**) Histograms of acidity and corresponding ratios of LASs and BASs in Ni_1_Co_5_O. (**e**) FTIR spectra of Ni_5_Co_1_O after pyridine adsorption. (**f**) Histograms of acidity and corresponding ratios of LASs and BASs in Ni_5_Co_1_O


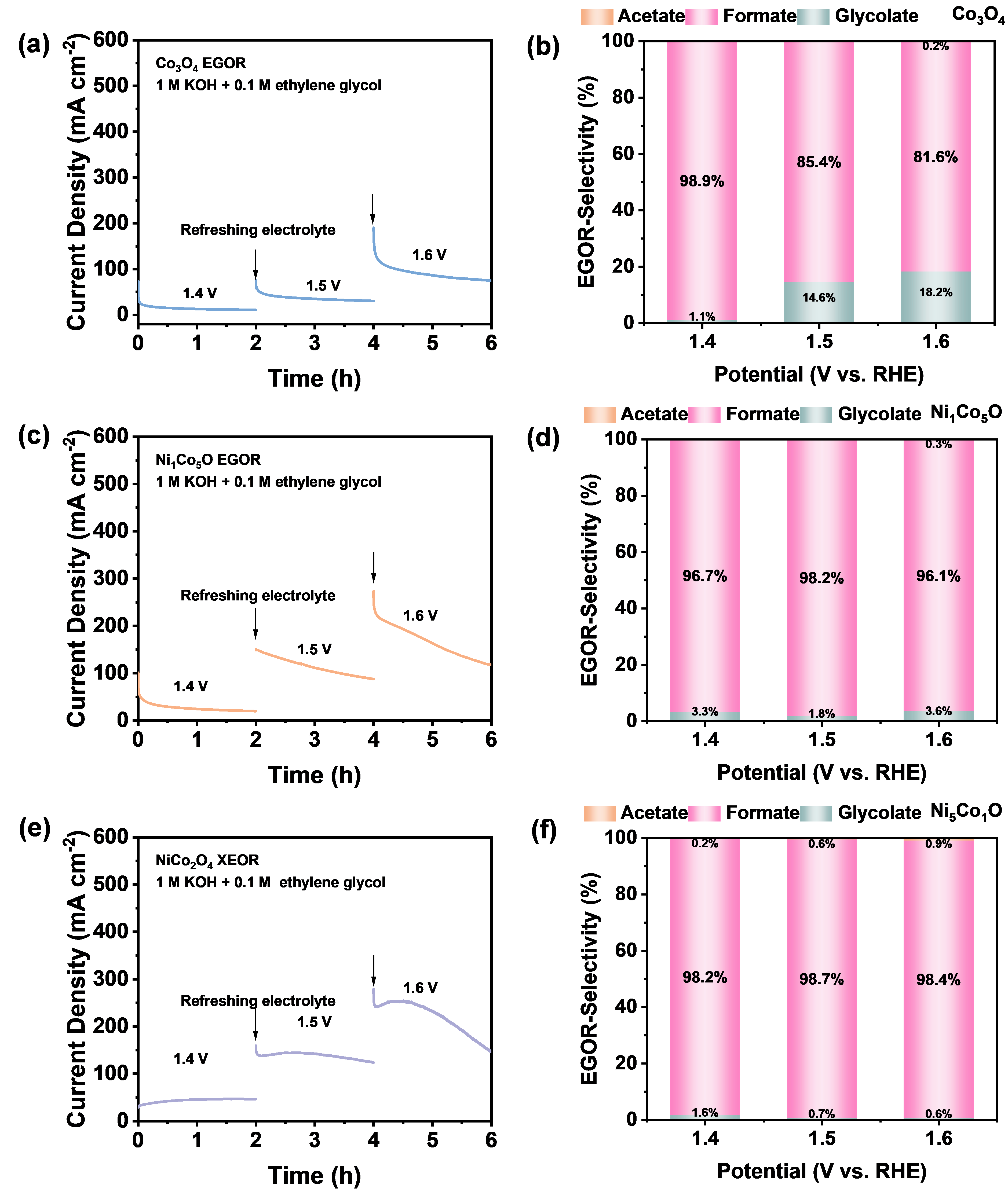


**Fig. S23** The i-t curves at constant applied potential of (**a**) Co_3_O_4_, (**c**) Ni_1_Co_5_O and (**e**) Ni_5_Co_1_O for product analysis. Product selectivity of (**b**) Co_3_O_4_, (**d**) Ni_1_Co_5_O and (**f**) Ni_5_Co_1_O for the oxidation of ethylene glycol at 1.4 V, 1.5 V, and 1.6 V (vs. RHE) for 2 h


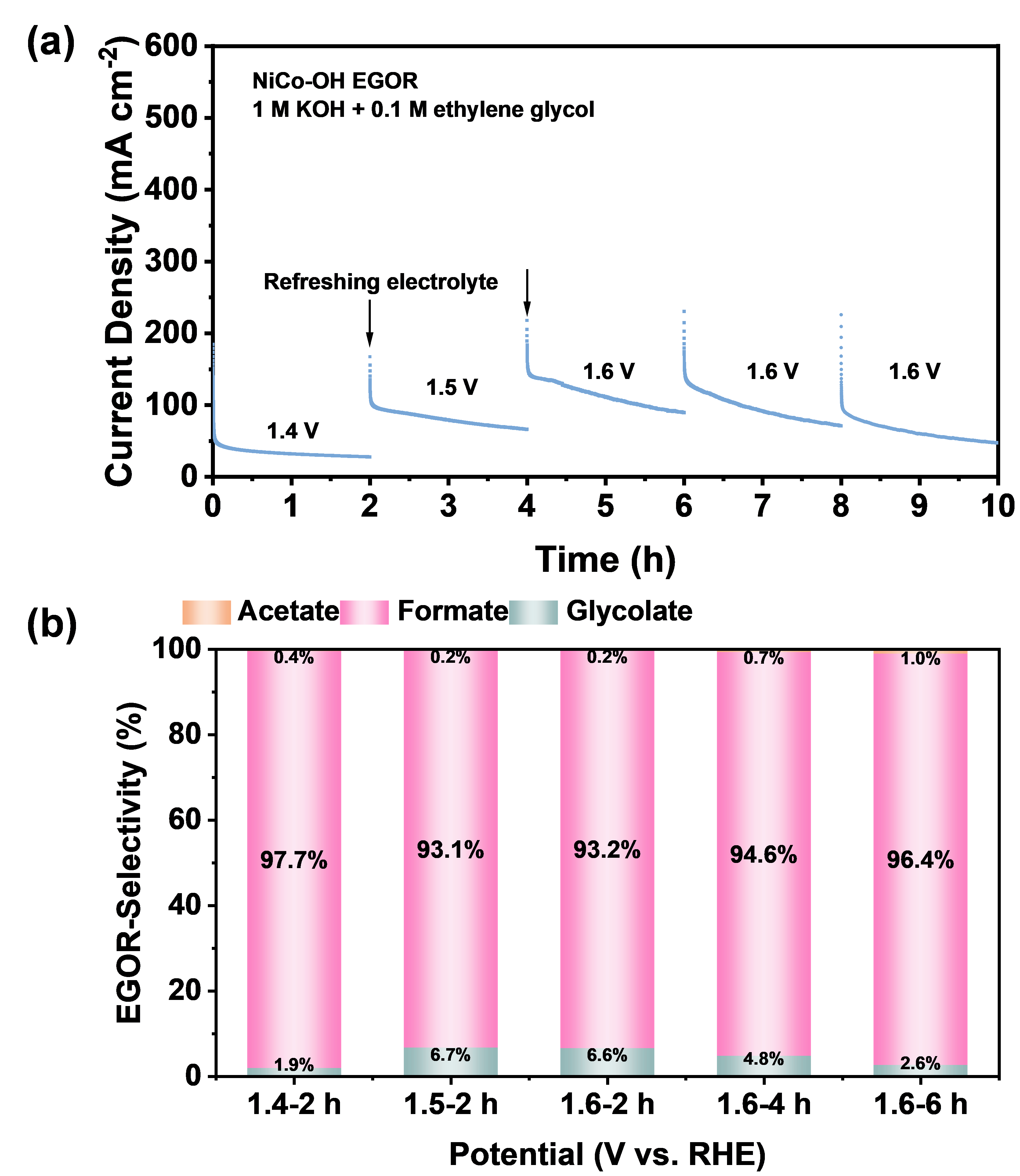


**Fig. S24** The i-t curves at constant applied potential of NiCoO-OH for product analysis. Product selectivity of NiCoO-OH for the oxidation of ethylene glycol

We successfully synthesized several oxide materials with different compositions, including Co_3_O_4_, Ni_1_Co_5_O, and Ni_5_Co_1_O, by precisely regulating the feeding ratio of materials. Through pyridine infrared tests (Table S4 and Fig. S22), it was found that these materials exhibited different ratios of BASs and LAS. Specifically, the proportion of L acid sites in Ni_1_Co_5_O was the highest, reaching 88.3%; Co_3_O_4_ contained 84.5% of LASs and 15.5% of BASs; while Ni_5_Co_1_O contained 79.7% of LASs and 20.3% of BASs. The selective performance of these materials in the ethylene glycol electrocatalytic reaction was further studied. The experimental conditions were to react for 2 h at 1.4 V, 1.5 V, and 1.6 V. The results showed (Fig. S23) that the selectivity of these three materials was lower than that of NiCo_2_O_4_ (Fig. S30a). Meanwhile, in order to further improve the manuscript, we supplemented the i-t test of NiCo-OH for ethylene glycol and its product analysis (as shown in Fig. S24). The results show that the formic acid selectivity of NiCo-OH in this reaction is still lower than that of NiCo_2_O_4_.To sum up, the above results fully demonstrated that NiCo_2_O_4_ showed the highest selectivity for formate due to its balanced BASs/LASs ratio.


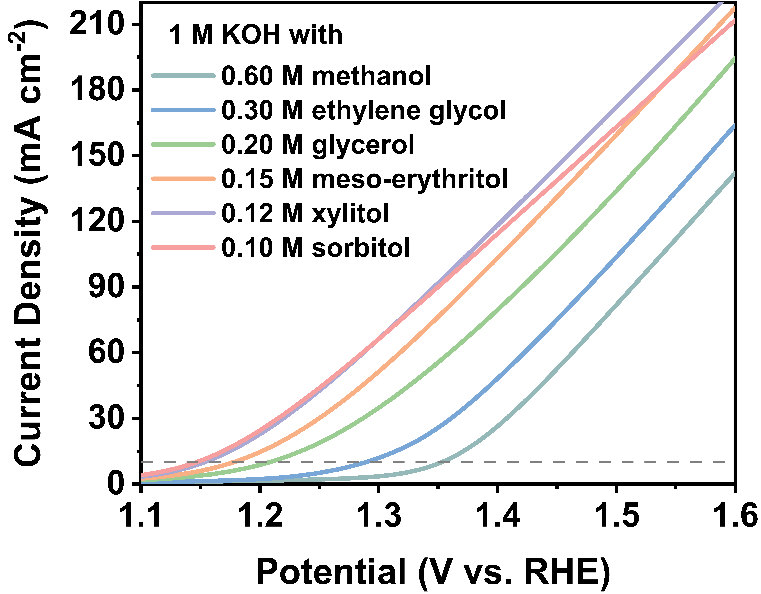


**Fig. 25** LSV curves (without iR-correction) of NiCo_2_O_4_ in 1 M KOH with X M C_1_-C_6_ saturated alcohol (e.g., 0.6 M methanol, 0.3 M ethylene glycol, 0.2 M glycerol, 0.15 M meso-erythritol, 0.12 M xylitol, and 0.1 M sorbitol) at a scan rate of 5 mV s^-1^

For the activity test of AOR of electrocatalytic C_1_-C_6_ saturated alcohols, we studied the performance variation law by controlling the consistency of the number of hydroxyl groups. Although the concentrations of SOR and XOR were relatively low, their potentials at 10 mA cm^-2^ (1.147 V and 1.154 V, respectively) were still significantly lower than those of MOR (1.353 V), EGOR (1.289 V), GOR (1.208 V), and MEOR (1.178 V). This result further verified the promoting effect of the increase in the number of hydroxyl groups on the catalytic activity. Specifically, the electrolytes used in the experiments were 1 M KOH + 0.6 M methanol, 0.3 M ethylene glycol, 0.2 M glycerol, 0.15 M meso-erythritol, 0.12 M xylitol, and 0.1 M sorbitol, respectively. To avoid errors introduced by operating conditions, nickel foam with a size of 1×1 cm^2^ was uniformly used as the electrode in this experiment, and the 1 M KOH solution prepared in the same batch was used. The results show that the higher the hydroxyl density in the saturated alcohol, the more significantly it can enhance the nucleophilic attack and adsorption, thereby improving the reaction activity. This finding further validates the key role of hydroxyl groups of saturated alcohol in the AOR reaction. In addition, in the 1 M KOH + 0.1 M sorbitol system, when the potential reached 1.4 V, the performance gradually decreased, which may be related to the higher difficulty of C-C bond cleavage.


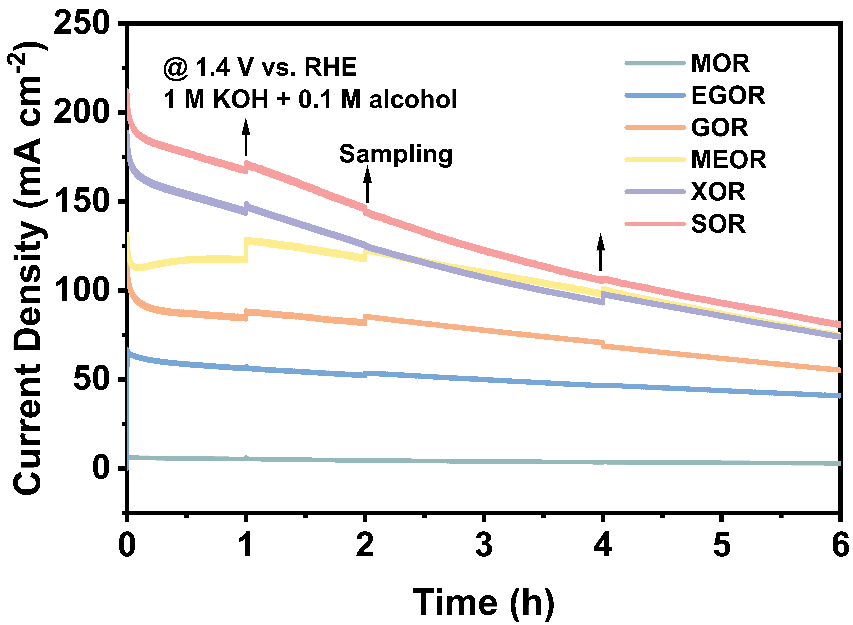


**Fig. S26** The i-t curves at a constant applied potential of 1.4 V (vs. RHE) in 40 mL electrolyte of 1 M KOH with 0.1 M alcohol for product analysis. 1 mL electrolyte was taken at 1 h, 2 h, 4 h and 6 h for HPLC testing


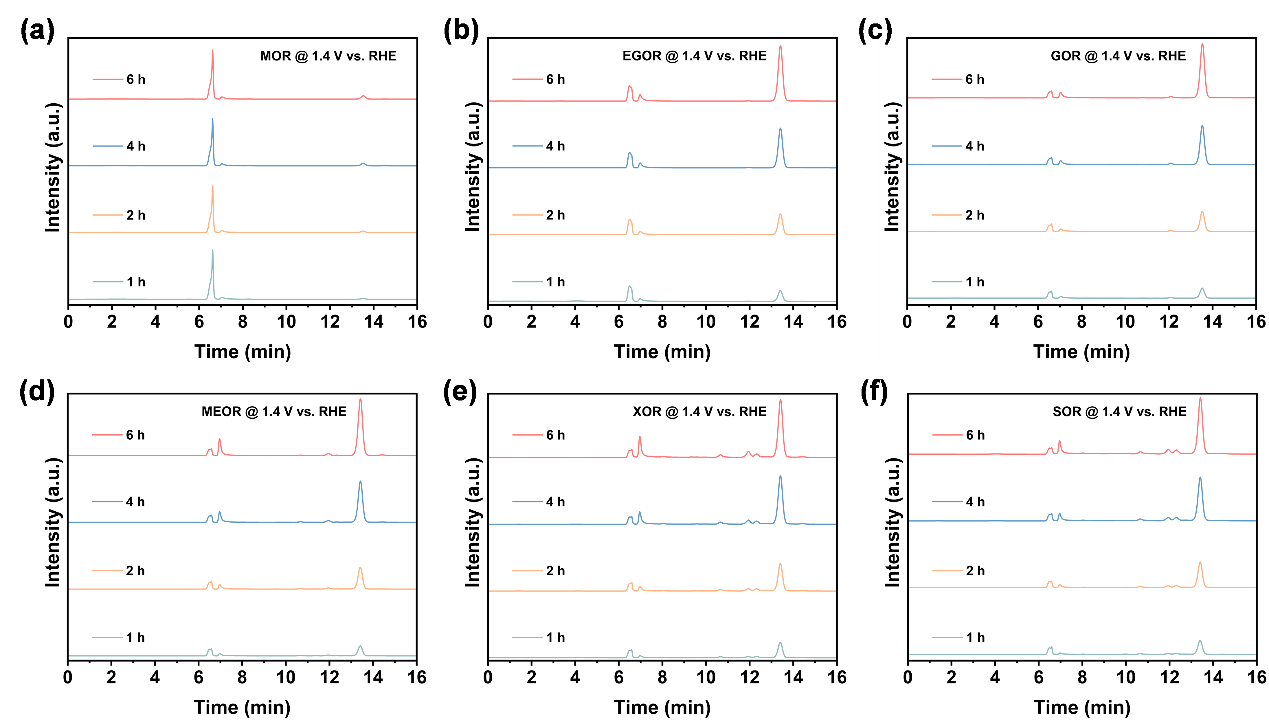


**Fig. S27** The evolution of HPLC chromatograms as a function of electrolyzing time. (**a**) MOR, (**b**) EGOR, (**c**) GOR, (**d**) MEOR, (**e**) XOR, and (**f**) SOR. The electrolysis experiments were carried out at a constant applied potential of 1.4 V (vs. RHE) in 40 mL electrolyte of 1 M KOH with 0.1 M alcohol for 6 h


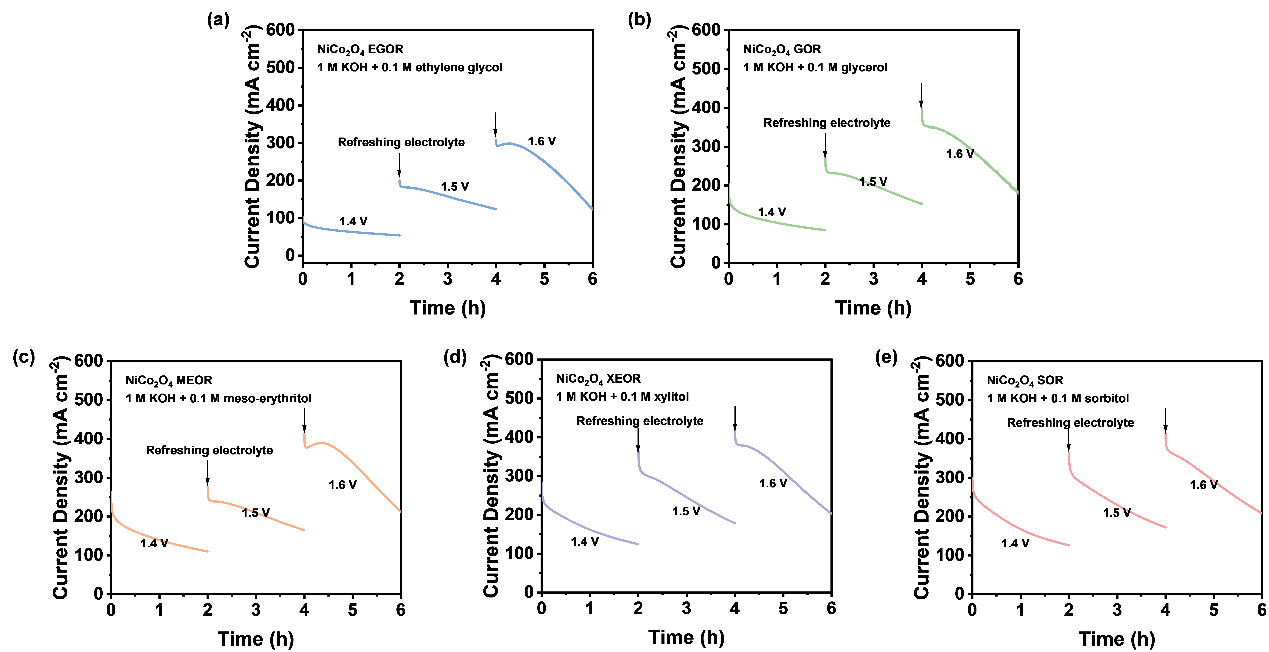


**Fig. S28** The i-t curves at constant applied potential of 1.4 V, 1.5 V, and 1.6 V (vs. RHE) in 40 mL electrolyte of 1 M KOH with 0.1 M alcohol for product analysis


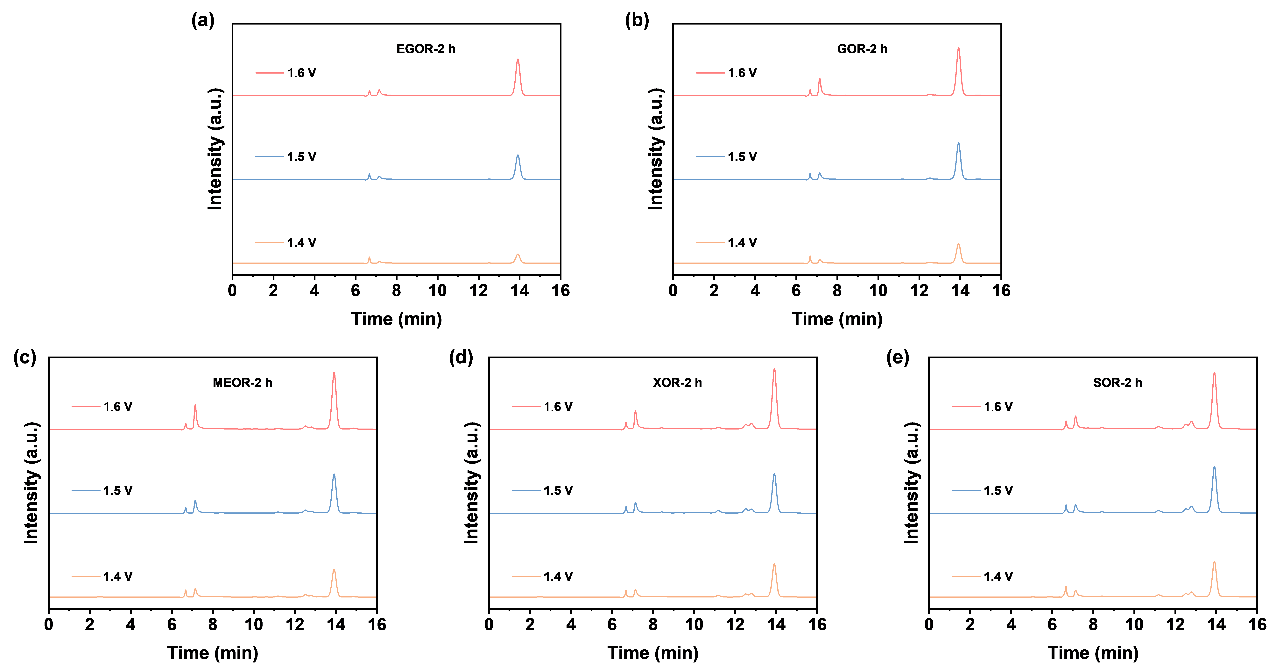


**Fig. S29** The evolution of HPLC chromatograms as a function of electrolyzing time. (**a**) EGOR, (**b**) GOR, (**c**) MEOR, (**d**) XOR, and (**e**) SOR. The electrolysis experiments were carried out at constant applied potential of 1.4 V, 1.5 V, and 1.6 V (vs. RHE) in 40 mL electrolyte of 1 M KOH with 0.1 M alcohol for 2 h


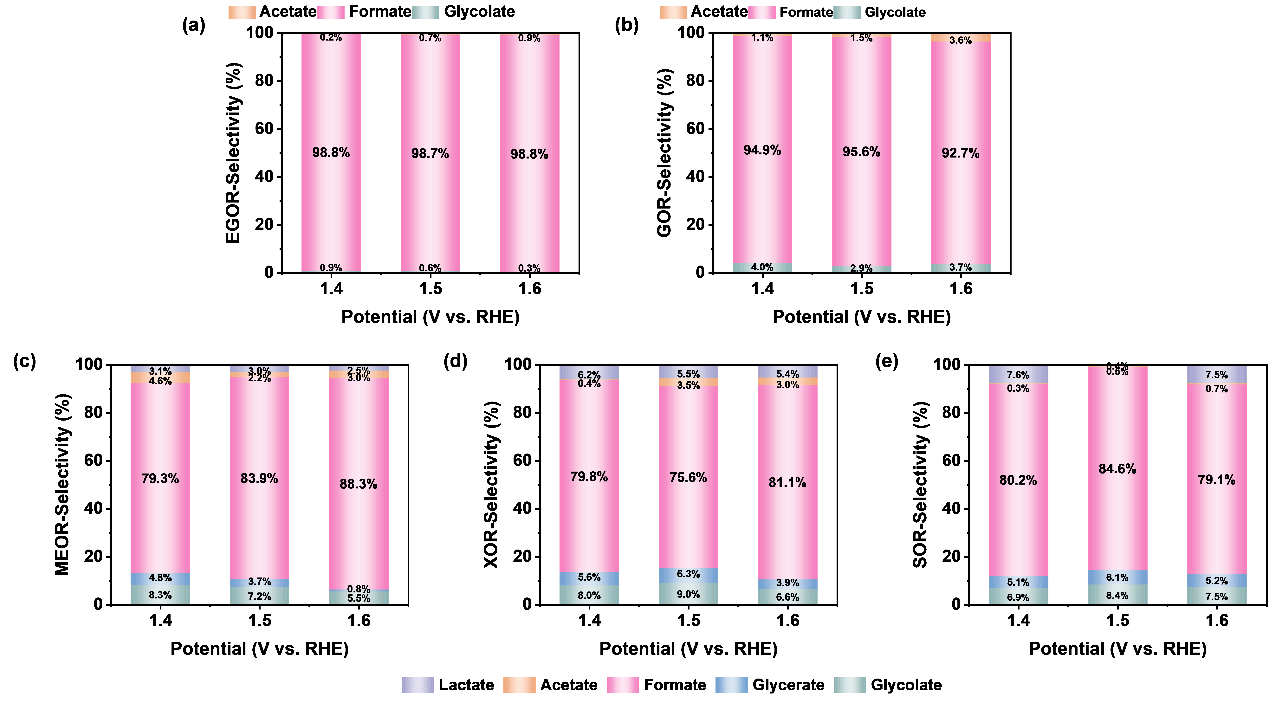


**Fig. S30** Product selectivity for the oxidation of (**a**) ethylene glycol, (**b**) glycerol, (**c**) meso-erythritol, (**d**) xylitol, and (**e**) sorbitol at 1.4 V, 1.5 V, and 1.6 V (vs. RHE) for 2 h

For each AOR, i-t tests were conducted for 2 h at potentials of 1.4 V, 1.5 V, and 1.6 V vs. RHE (Fig. S28). Subsequently, liquid chromatography analysis was performed on the post-reaction solutions (Fig. S29). As shown in Fig. S30, the selectivity results indicated that formic acid was the main product, and the more hydroxyl groups in the saturated alcohol molecule, the lower the selectivity of formic acid. Specifically, at a potential of 1.6 V vs. RHE, the selectivity of formate generated from the oxidation reactions of ethylene glycol, meso-erythritol, and xylitol were 98.8%, 88.3%, and 81.1%, respectively; while at a potential of 1.5 V vs. RHE, the selectivity of formate generated from the oxidation reactions of glycerol and sorbitol were the highest, being 95.6% and 84.6%, respectively.


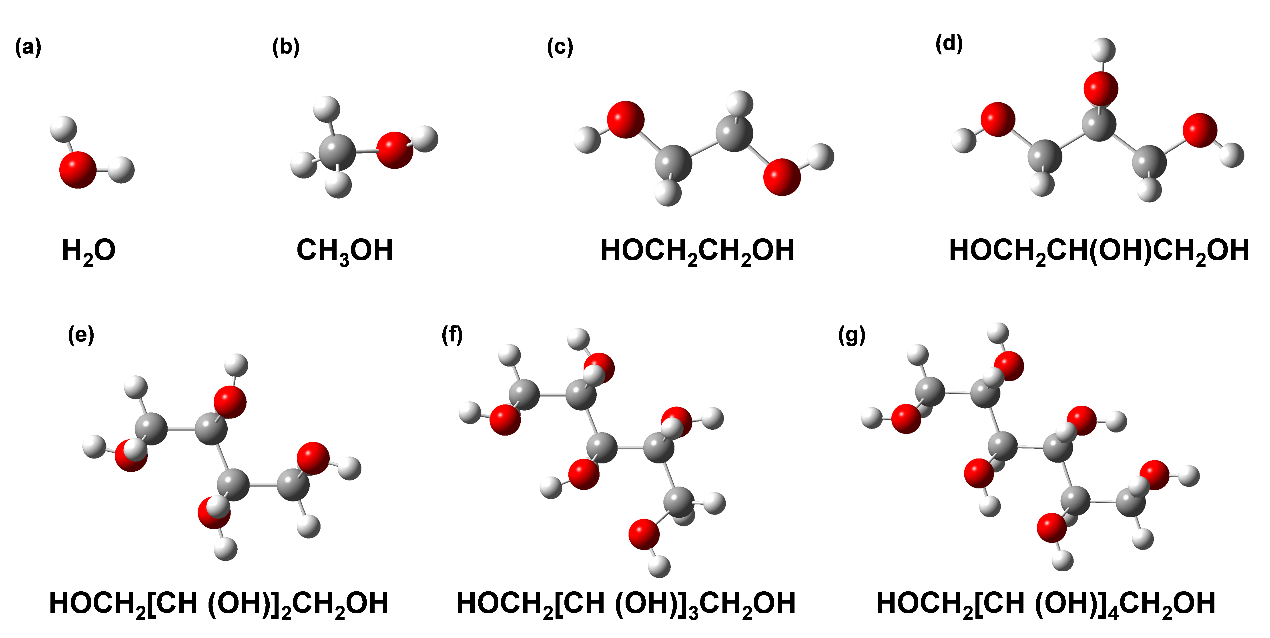


**Fig. S31** Initial model of (**a**) water, (**b**) methanol, (**c**) ethylene glycol, (**d**) glycerol, (**e**) meso-erythritol, (**f**) xylitol, and (**g**) sorbitol molecules constructed using Gaussian 09W


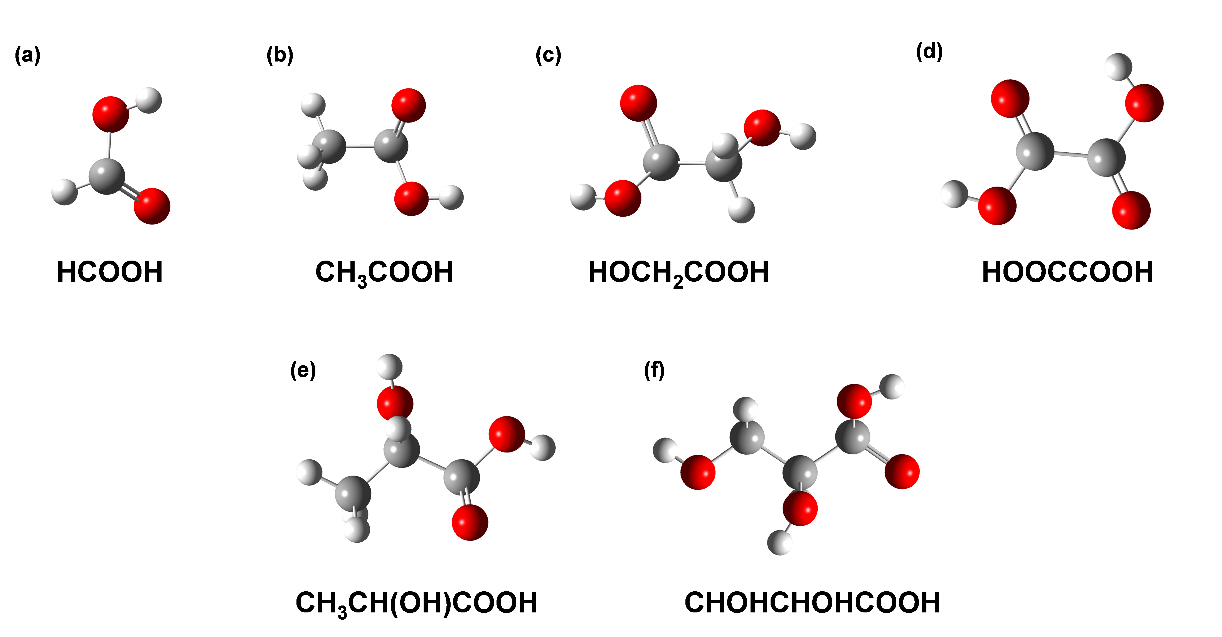


**Fig. S32** Initial model of (**a**) formate, (**b**) acetate, (**c**) glycolate, (**d**) oxalate, (**e**) lactate, and (**f**) glycerate molecules constructed using Gaussian 09W


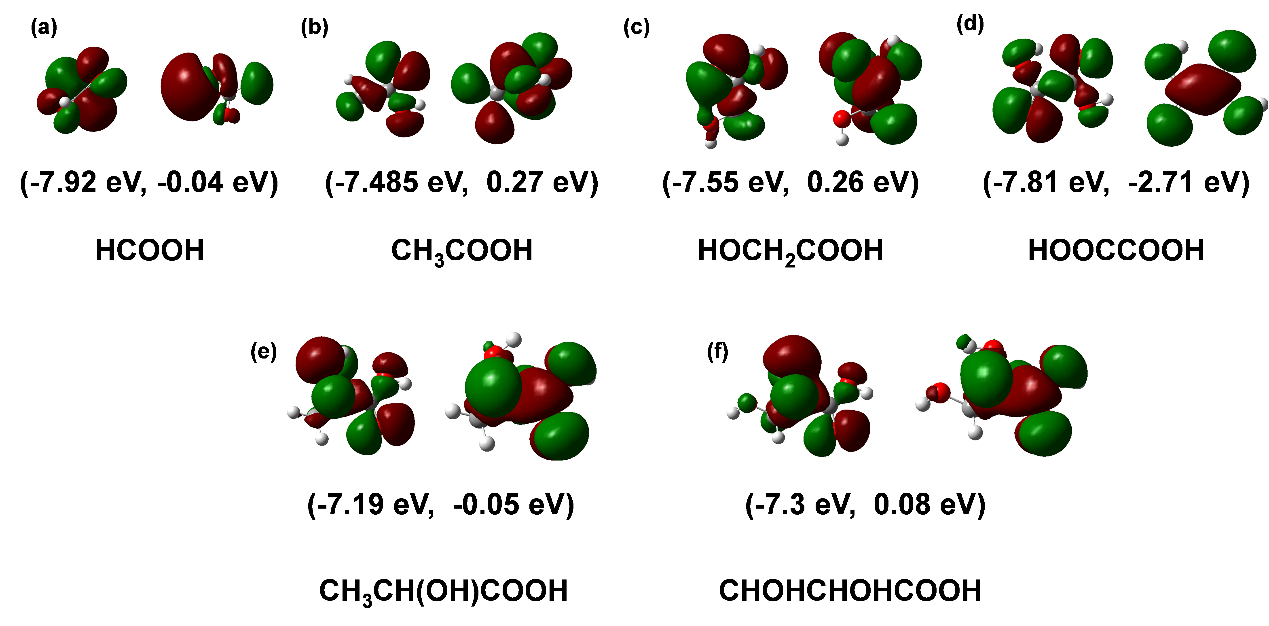


**Fig. S33** HOMO orbitals (left) and LUMO (right) orbitals of (**a**) formate, (**b**) acetate, (**c**) glycolate, (**d**) oxalate, (**e**) lactate, and (**f**) glycerate molecules constructed using Gaussian 09W


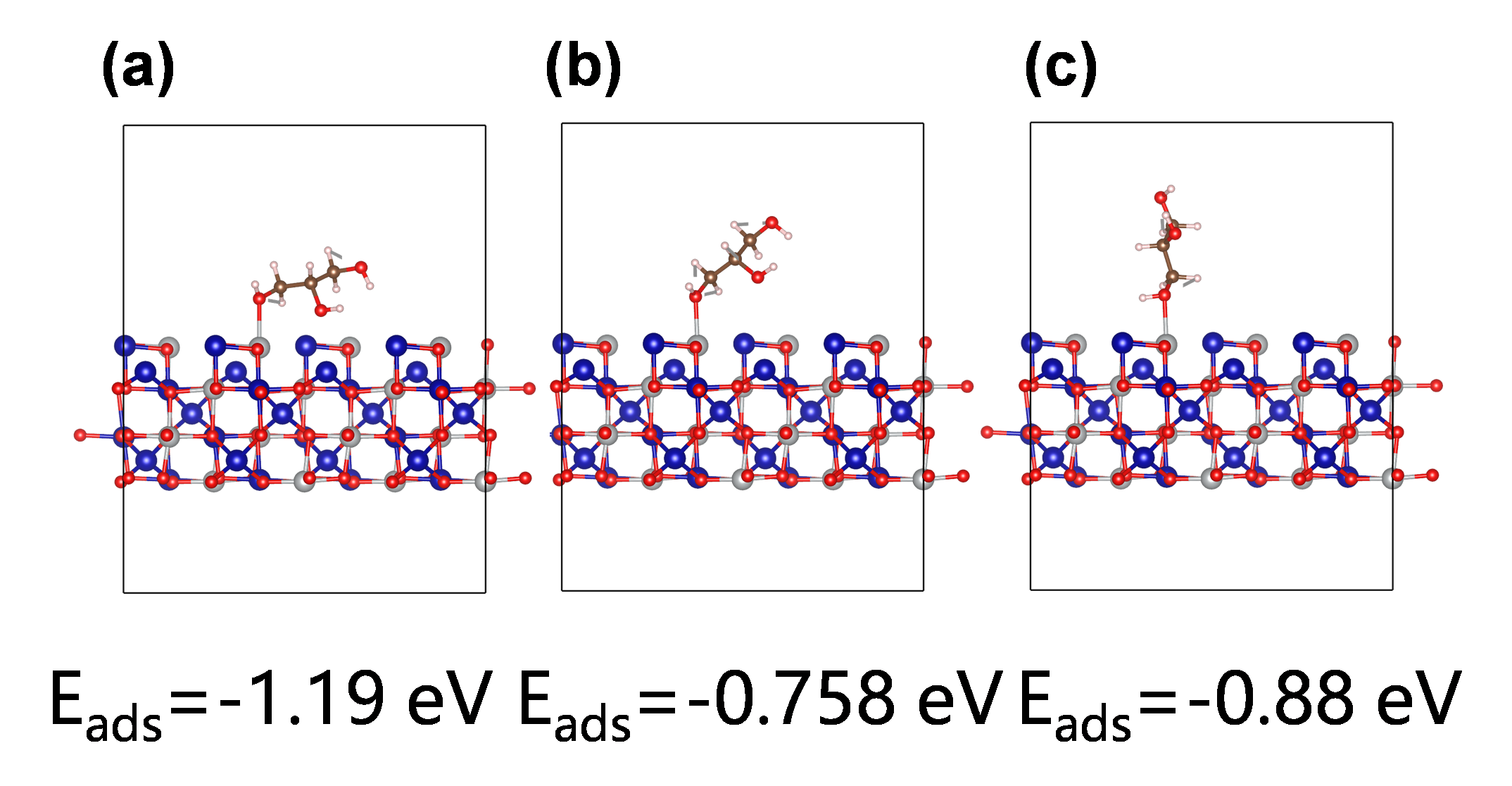


**Fig. S34** The adsorption energies of glycerol molecules placed (**a**) horizontally, (**b**) obliquely and (**c**) vertically on the surface of NiCo_2_O_4_ (110)

Glycerol molecules were adsorbed on the NiCo_2_O_4_ (110) surface in three ways: horizontally, obliquely and vertically, respectively. The calculated adsorption energies were -1.19 eV, -0.758 eV and -0.88 eV, respectively (Fig. S1). Among them, the vertical adsorption exhibited a moderate adsorption energy. Therefore, in the subsequent assessment of adsorption capacity, it was chosen to place the alcohol molecules in a vertical configuration on the NiCo_2_O_4_ (110) surface.

**Table S1** Comparison of AOR activity of NiCo-based hydroxides and oxides electrocatalysts, published recently

| **Sample ID** | **Current density**  **(mA cm^-2^)** | | **Potential**  **(V vs. RHE)** | **Electrolyte** | **Refs.** |
| --- | --- | --- | --- | --- | --- |
| CoNi LDH | | 10 | 1.36 | 1.0 M KOH + 0.15 M glycerol | ACS Appl. Mater. Interfaces [S1] |
| NiCo hydroxide | | 100 | 1.35 | 1.0 M KOH + 0.10 M glycerol | Nat. Commun. **[**S2] |
| NiVRu-LDHs NAs/NF | | 10 | 1.35 V | 1.0 M KOH + 1.0 M glycerol | Adv. Mater. [S3] |
| Mn-Co (hydroxide)-S/NF | | 100 | 1.347 | 1.0 M KOH + 0.10 M glycerol | Nano Energy [S4] |
| NiOx/MWCNTs-Ox | | 10 | 1.31 | 1.0 M KOH + 0.1 M glycerol | ACS Catal. [S5] |
| NiCrO-V_Cr,O_ nanosheets | | 10 | 1.37 | 1.0 M KOH + 0.1 M glycerol | ACS Catal. [S6] |
| Bi-Co_3_O_4_ | | 100 | 1.297 | 1.0 M KOH + 0.1 M glycerol | ACS Catal. [S7] |
| CoO/CFP | | 10 | 1.32 V | 1.0 M KOH + 0.1 M glycerol | Adv. Energy Mater. [S8] |
| NiCo_2_O_4_/NF | | 10 | 1.13 | 1.0 M KOH + 0.1 M glycerol | Adv. Funct. Mater. [S9] |
| NiCo_2_O_4_/NF | | 10 | 1.219 | 1.0 M KOH + 0.1 M glycerol | Angew. Chem. Int. Ed. [S10] |
| NiCo_2_O_4_ nanosheets | | 10 | 1.23 | 1.0 M LiOH + 0.1 M glycerol | Chin. J. Catal. [S11] |
| CuCo_2_O_4−x_-2 | | 10 | 1.266 | 0.1 M KOH + 0.1 M glycerol | Adv. Mater. [S12] |
| CuCo-oxide | | 10 | 1.25 | 0.1 M KOH + 0.1 M glycerol | Adv. Mater. [S13] |
| R-NiCuO | | 10 | 1.2 | 1.0 M LiOH + 0.1 M glycerol | Energy Environ. Sci. [S14] |

**Table S2**. Comparison of OER activity of NiCo-based hydroxides and oxides electrocatalysts in 1 M KOH, published recently

| **Sample ID** | **Current density**  **(mA cm^-2^)** | **Potential** | **Tafel slope**  **(mV dec^-1^)** | **Refs.** |
| --- | --- | --- | --- | --- |
| Ni_x_Fe_y_Co_z_(OH)_m_ | 10 | 146 mV | 50 | Energy Environ. Sci. [S15] |
| Co(OH)_2_/NiP_x_ | 10 | 236 mV | 52 | Adv. Funct. Mater. [S16] |
| Mo1-NiFeOxHy | 10 | 193 mV | 32.33 | Adv. Mater. [S17] |
| Se-(NiCo)S/(OH) | 10 | 158 mV | 31.9 | Adv. Mater. [S18] |
| d-NiFe-LDH | 10 | 170 mV | 24.9 | Angew. Chem. Int. Ed. [S19] |
| Fe-NiCo-LDH | 50 | 234 mV | 51.9 | Adv. Funct. Mater. [S20] |
| NiFe/NiFe−OH | 10 | 222 mV | 41 | Appl. Catal. B-Environ [S21] |
| NiCo_2-x_Fe_x_O_4_ NBs | 10 | 274 mV | 42 | Angew Chem Int Ed.[S22] |
| NiFe_2_O_4_-S-2 | 10 | 180 mV | 62.6 | J. Am. Chem. Soc.[S23] |
| NiFeO_x_-P | 10 | 237 mV | 27.07 | Angew Chem Int Ed.[S24] |
| NiCo_1.8_Fe_0.2_O_4_@N-carbon | 10 | 270 mV | 130 | Angew Chem Int Ed.[S25] |
| 0.5Fe-NiCo_2_O_4_ | 10 | 258 mV | 63.5 | Small [S26] |
| NiCo@NiCoO_2_/C PMRAs | 20 | 366 mV | 83.97 | Adv. Mater. [S27] |

**Table S3** The concentrations of Lewis acid sites (LASs) and Brønsted acid sites (BASs) in NiCo-OH and NiCo_2_O_4_.

|  | **T (^o^C)** | **BASs (mmol/g)** | **LASs (mmol/g)** |
| --- | --- | --- | --- |
| **NiCo_2_O_4_** | 150 | 0.05943808 | 0.06718517 |
| **NiCo-OH** | 150 | 0.02387788 | 0.002769 |

**Table S4** The concentrations of Lewis acid sites (LASs) and Brønsted acid sites (BASs) in other electrocatalysts of different proportions

|  | **T (^o^C)** | **BASs (mmol/g)** | **LASs (mmol/g)** |
| --- | --- | --- | --- |
| **Co_3_O_4_** | 150 | 0.02041 | 0.11093 |
| **Ni_1_Co_5_O** | 150 | 0.01549 | 0.11711 |
| **Ni_5_Co_1_O** | 150 | 0.02208 | 0.08651 |

***Ni_1_Co_5_O, feed molar ratio: 1 (**Ni(NO_3_)_2_·6H_2_O**):5 (**Co(NO_3_)_2_·6H_2_O**).**

***Ni_5_Co_1_O, feed molar ratio: 5 (**Ni(NO_3_)_2_·6H_2_O**):1 (**Co(NO_3_)_2_·6H_2_O**).**

**Supplementary References**

1. N. Shilpa, A. Pandikassala, P. Krishnaraj, P. S. Walko, R. N. Devi et al., Co-Ni Layered Double Hydroxide for the Electrocatalytic Oxidation of Organic Molecules: An Approach to Lowering the Overall Cell Voltage for the Water Splitting Process. ACS Appl. Mater. Interfaces **14**, 16222-16232 (2022). <https://doi.org/10.1021/acsami.2c00982>
2. Z. He, J. Hwang, Z. Gong, M. Zhou, N. Zhang et al., Promoting biomass electrooxidation via modulating proton and oxygen anion deintercalation in hydroxide. Nat. Commun. **13**, 3777 (2022). <https://doi.org/10.1038/s41467-022-31484-0>
3. Q. Qian, X. He, Z. Li, Y. Chen, Y. Feng et al., Electrochemical Biomass Upgrading Coupled with Hydrogen Production under Industrial-level Current Density. Adv. Mater. **35**, e2300935 (2023). <https://doi.org/10.1002/adma.202300935>
4. Y. Fang, C. Dai, X. Liu, Y. Wang, C. Ju et al., Sulfur-doped manganese-cobalt hydroxide with promoted surface reconstruction for glycerol electrooxidation assisted hydrogen production. Nano Energy **127**, 109754 (2024). [https://doi.org/10.1016/j.nanoen.2024.109754](https://doi.org/https://doi.org/10.1016/j.nanoen.2024.109754)
5. D. M. Morales, D. Jambrec, M. A. Kazakova, M. Braun, N. Sikdar et al., Electrocatalytic Conversion of Glycerol to Oxalate on Ni Oxide Nanoparticles-Modified Oxidized Multiwalled Carbon Nanotubes. ACS Catal. **12**, 982-992 (2022). <https://doi.org/10.1021/acscatal.1c04150>
6. Z. Xia, C. Ma, Y. Fan, Y. Lu, Y.-C. Huang et al., Vacancy Optimized Coordination on Nickel Oxide for Selective Electrocatalytic Oxidation of Glycerol. ACS Catal. **14**, 1930-1938 (2024). <https://doi.org/10.1021/acscatal.3c04568>
7. Y. Wang, Y.-Q. Zhu, Z. Xie, S.-M. Xu, M. Xu et al., Efficient Electrocatalytic Oxidation of Glycerol via Promoted OH* Generation over Single-Atom-Bismuth-Doped Spinel Co_3_O_4_. ACS Catal. **12**, 12432-12443 (2022). <https://doi.org/10.1021/acscatal.2c03162>
8. N. Xi, Y. Zang, X. Sun, J. Yu, M. Johnsson et al., Polyhedral Coordination Determined Co‐O Activity for Electrochemical Oxidation of Biomass Alcohols. Adv. Energy Mater. **13**, 2301572 (2023). <https://doi.org/10.1002/aenm.202301572>
9. W. Luo, H. Tian, Q. Li, G. Meng, Z. Chang et al., Controllable Electron Distribution Reconstruction of Spinel NiCo_2_O_4_ Boosting Glycerol Oxidation at Elevated Current Density. Adv. Funct. Mater. **34**, 2306995 (2023). <https://doi.org/10.1002/adfm.202306995>
10. J. Ma, X. Wang, J. Song, Y. Tang, T. Sun et al., Synergistic Lewis and Brønsted Acid Sites Promote OH* Formation and Enhance Formate Selectivity: Towards High-efficiency Glycerol Valorization. Angew. Chem. Int. Edl **63**, e202319153 (2024). <https://doi.org/10.1002/anie.202319153>
11. Y. Duan, M. Xue, B. Liu, M. Zhang, Y. Wang et al., Integration of theory prediction and experimental electrooxidation of glycerol on NiCo_2_O_4_ nanosheets. Chinese J. Catal. **57**, 68-79 (2024). https://doi.org/10.1016/S1872-2067(23)64585-1
12. L. Wu, Q. Wu, Y. Han, D. Zhang, R. Zhang et al., Strengthening the Synergy between Oxygen Vacancies in Electrocatalysts for Efficient Glycerol Electrooxidation. Adv. Mater. **36**, 2401857 (2024). <https://doi.org/10.1002/adma.202401857>
13. L. S. Oh, M. Park, Y. S. Park, Y. Kim, W. Yoon et al., How to Change the Reaction Chemistry on Nonprecious Metal Oxide Nanostructure Materials for Electrocatalytic Oxidation of Biomass-Derived Glycerol to Renewable Chemicals. Adv. Mater. **35**, e2203285 (2023). <https://doi.org/10.1002/adma.202203285>
14. S. Li, P. Ma, C. Gao, L. Liu, X. Wang et al., Reconstruction-induced NiCu-based catalysts towards paired electrochemical refining. Energy Environ. Sci. **15**, 3004-3014 (2022). <https://doi.org/10.1039/d2ee00461e>
15. D. Zhang, H. Li, H. Lu, Z. Yin, Z. Fusco et al., Unlocking the performance of ternary metal (hydro)oxide amorphous catalysts via data-driven active-site engineering. Energy Environ. Sci. **16**, 5065-5075 (2023). <https://doi.org/10.1039/D3EE01981K>
16. M. Chen, H. Li, C. Wu, Y. Liang, J. Qi et al., Interfacial Engineering of Heterostructured Co(OH)_2_/NiP_x_ Nanosheets for Enhanced Oxygen Evolution Reaction. Adv. Funct. Mater. **32**, 2206407 (2022). <https://doi.org/10.1002/adfm.202206407>
17. Y. Wu, Y. Zhao, P. Zhai, C. Wang, J. Gao et al., Triggering Lattice Oxygen Activation of Single-Atomic Mo Sites Anchored on Ni–Fe Oxyhydroxides Nanoarrays for Electrochemical Water Oxidation. Adv. Mater. **34**, 2202523 (2022). https://doi.org/10.1002/adma.202202523
18. C. Hu, L. Zhang, Z.-J. Zhao, A. Li, X. Chang et al., Synergism of Geometric Construction and Electronic Regulation: 3D Se-(NiCo)S/(OH) Nanosheets for Highly Efficient Overall Water Splitting. Adv. Mater. **30**, 1705538 (2018). https://doi.org/10.1002/adma.201705538
19. Z. Li, Y. Zhou, M. Xie, H. Cheng, T. Wang et al., High-Density Cationic Defects Coupling with Local Alkaline-Enriched Environment for Efficient and Stable Water Oxidation. Angew. Chem. Int. Ed. **62**, e202217815 (2023). https://doi.org/10.1002/anie.202217815
20. H. Zhang, H. Guo, Y. Li, Q. Zhang, L. Zheng et al., The Guest Doping Effects of Fe on Bimetallic NiCo Layered Double Hydroxide for Enhanced Electrochemical Oxygen Evolution Reaction: Theoretical Screening and Experimental Verification. Adv. Funct. Mater. **33**, 2304403 (2023). https://doi.org/10.1002/adfm.202304403
21. W. Zhu, W. Chen, H. Yu, Y. Zeng, F. Ming et al., NiCo/NiCo–OH and NiFe/NiFe–OH core shell nanostructures for water splitting electrocatalysis at large currents. Appl. Catal. B: Environ. **278**, 119326 (2020). <https://doi.org/10.1016/j.apcatb.2020.119326>
22. Y. Huang, S. L. Zhang, X. F. Lu, Z. P. Wu, D. Luan et al., Trimetallic Spinel NiCo_2-x_Fe_x_O_4_ Nanoboxes for Highly Efficient Electrocatalytic Oxygen Evolution. Angew. Chem. Int. Ed. **60**, 11841-11846 (2021). <https://doi.org/10.1002/anie.202103058>
23. Y. Yan, J. Lin, K. Huang, X. Zheng, L. Qiao et al., Tensile Strain-Mediated Spinel Ferrites Enable Superior Oxygen Evolution Activity. J. Am. Chem. Soc. **145**, 24218-24229 (2023). <https://doi.org/10.1021/jacs.3c08598>
24. X. Li, C. Deng, Y. Kong, Q. Huo, L. Mi et al., Unlocking the Transition of Electrochemical Water Oxidation Mechanism Induced by Heteroatom Doping. Angew. Chem. Int. Ed. **62**, e202309732 (2023). https://doi.org/10.1002/anie.202309732
25. Y. Liu, L. Zhou, S. Liu, S. Li, J. Zhou et al., Fe, N‐Inducing Interfacial Electron Redistribution in NiCo Spinel on Biomass‐Derived Carbon for Bi‐functional Oxygen Conversion. Angew. Chem. Int. Ed. **63**, e202319983 (2024). <https://doi.org/10.1002/anie.202319983>
26. J. Yang, Y. Wang, J. Yang, Y. Pang, X. Zhu et al., Quench‐Induced Surface Engineering Boosts Alkaline Freshwater and Seawater Oxygen Evolution Reaction of Porous NiCo_2_O_4_ Nanowires. Small **18**, 2106187 (2021). <https://doi.org/10.1002/smll.202106187>
27. H. Xu, Z. X. Shi, Y. X. Tong & G. R. Li. Porous Microrod Arrays Constructed by Carbon‐Confined NiCo@NiCoO_2_ Core@Shell Nanoparticles as Efficient Electrocatalysts for Oxygen Evolution. Adv. Mater. **30**, 1705442 (2018). <https://doi.org/10.1002/adma.201705442>
